# Dosing pole recommendations for lymphatic filariasis elimination: A height-weight quantile regression modeling approach

**DOI:** 10.1371/journal.pntd.0007541

**Published:** 2019-07-17

**Authors:** Charles W. Goss, Katiuscia O’Brian, Christine Dubray, Peter U. Fischer, Myra Hardy, Purushothaman Jambulingam, Christopher L. King, Moses Laman, Jean Frantz Lemoine, Leanne J. Robinson, Josaia Samuela, Swaminathan Subramanian, Taniawati Supali, Gary J. Weil, Kenneth B. Schechtman

**Affiliations:** 1 Washington University, St. Louis, Missouri, United States of America; 2 Centers of Disease Control and Prevention, Atlanta, Georgia, United States of America; 3 Murdoch Children’s Research Institute, Melbourne, Australia; 4 The University of Melbourne, Melbourne, Australia; 5 ICMR-Vector Control Research Centre, Puducherry, India; 6 Case Western Reserve University, Cleveland, Ohio, United States of America; 7 Papua New Guinea Institute of Medical Research, Madang, Papua New Guinea; 8 Ministère de la Santé Publique et de la Population (MSPP), Port au Prince, Haiti; 9 Burnet Institute, Melbourne, Australia; 10 Fiji Ministry of Health and Medical Services, Suva, Fiji; 11 Universitas Indonesia, Jakarta, Indonesia; Faculty of Science, Ain Shams University (ASU), EGYPT

## Abstract

**Background:**

The World Health Organization (WHO) currently recommends height or age-based dosing as alternatives to weight-based dosing for mass drug administration lymphatic filariasis (LF) elimination programs. The goals of our study were to compare these alternative dosing strategies to weight-based dosing and to develop and evaluate new height-based dosing pole scenarios.

**Methodology/Principal findings:**

Age, height and weight data were collected from >26,000 individuals in five countries during a cluster randomized LF clinical trial. Weight-based dosing for diethylcarbamazine (DEC; 6 mg/kg) and ivermectin (IVM; 200 ug/kg) with tablet numbers derived from a table of weight intervals was treated as the “gold standard” for this study. Following WHO recommended age-based dosing of DEC and height-based dosing of IVM would have resulted in 32% and 27% of individuals receiving treatment doses below those recommended by weight-based dosing for DEC and IVM, respectively. Underdosing would have been especially common in adult males, who tend to have the highest LF prevalence in many endemic areas. We used a 3-step modeling approach to develop and evaluate new dosing pole cutoffs. First, we analyzed the clinical trial data using quantile regression to predict weight from height. We then used weight predictions to develop new dosing pole cutoff values. Finally, we compared different dosing pole cutoffs and age and height-based WHO dosing recommendations to weight-based dosing. We considered hundreds of scenarios including country- and sex-specific dosing poles. A simple dosing pole with a 6-tablet maximum for both DEC and IVM reduced the underdosing rate by 30% and 21%, respectively, and was nearly as effective as more complex pole combinations for reducing underdosing.

**Conclusions/Significance:**

Using a novel modeling approach, we developed a simple dosing pole that would markedly reduce underdosing for DEC and IVM in MDA programs compared to current WHO recommended height or age-based dosing.

## Author summary

Lymphatic filariasis is a debilitating parasitic disease that affects over 65 million people globally. To eliminate this disease the World Health Organization (WHO) recommends treating entire communities with drug combinations that include ivermectin and diethylcarbamazine. Ideally, the amount of drug administered is determined by weight; however, obtaining accurate weight measurements in remote, resource limited areas is oftentimes not feasible. The alternatives currently recommended by the WHO based on height (ivermectin) and age (diethylcarbamazine) have maximal doses below that recommended by weight-based dosing. In this study we use statistical models, based on data from a large (5-country, >26,000 individuals) lymphatic filariasis clinical trial, to develop model-based dosing poles and compare dosing based on our dosing models to WHO recommendations. Our results showed that the WHO methods would have resulted in 32% (diethylcarbamazine) and 27% (ivermectin) of individuals in the clinical trial dataset to receive below the recommended weight-based dosage. Dosing poles based on our statistical models showed that our dosing pole would markedly reduce underdosing with 2% and 6% receiving below the recommended dosage for diethylcarbamazine and ivermectin, respectively. The dosing pole we propose has the potential to dramatically improve dosing and facilitate the elimination of lymphatic filariasis globally.

## Introduction

Lymphatic filariasis (LF) is a disabling mosquito-borne parasitic disease that affects some 68 million people globally [1]; the WHO estimated more than 880 million people in 51 countries remained at risk for LF in 2017 [2]. In the year 2000, the World Health Organization (WHO) implemented a strategic plan (Global Programme to Eliminate Lymphatic Filariasis [GPELF]) to eliminate LF as a public health problem by 2020 [3, 4]. As part of GPELF, the WHO recommends using two-drug treatment combinations (diethylcarbamazine [DEC] + albendazole or ivermectin [IVM] + albendazole) in mass drug administration (MDA) programs that dose both “at risk” and infected individuals in LF-endemic areas [3, 4]. Recent studies have shown that a triple-drug treatment combination (IVM + DEC + albendazole, IDA) is more effective [5] and as safe [6] as a standard two-drug LF treatment (DEC + albendazole, DA). This has resulted in an updated WHO policy to include IDA as an option for LF treatment in certain settings [7] which will help to more rapidly achieve the GPELF goal of eliminating LF as a global public health problem.

Key components of GPELF include the use of MDA to deliver treatment to infected people and to reduce parasite transmission by reducing the reservoir of parasites required for mosquito transmission. The WHO recommends weight-based dosing for IVM and DEC [3, 4]. However, this is often not possible in remote, resource-limited areas. Consequently, when weight-based dosing is not feasible, the WHO has recommended alternatives such as the use of height-based dosing poles for IVM and age-based dosing for DEC. The current height-based IVM dosing pole recommended by the WHO was developed in the early 1990s [8–10]; it was originally employed in Nigerian communities based on 150 μg/kg and has a dosing range of 3 to 12 mg for four different height groupings (90 to 119 cm, 120 to 140 cm, 141 to 158 cm, and > 158 cm). The WHO age-based dosing for DEC ranges from 100 to 300 mg across three different age groupings (2 to 5 years, 6 to 15 years, > 15 years) [9]. Although height and age-based dosing are recommended by the WHO for LF MDA programs, the maximum recommended doses using these methods are lower than those indicated by weight-based dosing (6 mg/kg for DEC and 200 μg/kg for IVM). Furthermore, having separate dosing methods for DEC and IVM increases the complexity and challenge of implementing the new 3-drug treatment for LF MDAs, and an alternative based on a single height pole for both DEC and IVM would increase the efficiency and feasibility of administering this new treatment option.

The primary objectives of our study were to: (1) compare the WHO’s height-(IVM) and age-(DEC) based dosing recommendations for MDA programs to gold-standard weight-based dosing with data from a variety of LF endemic areas; (2) to use field data to develop and evaluate alternative height-based dosing poles for IVM and DEC; and (3) determine whether a single height-based dosing pole can be used to administer both IVM and DEC with the aim of reducing underdosing compared to current WHO recommended methods. To address these objectives, we analyzed height and weight data collected as part of a large LF drug-safety clinical trial [6].

## Methods

### Ethics statement

The study protocols were reviewed and approved by independent Federal-Wide Assurance (FWA) registered ethical review boards in each country and at institutions of research partners who participated in the studies. The de-identified data used in this study were limited to gender, height, weight, age and country.

### Data source

The data were collected during a community-based safety study of MDA for LF that enrolled more than 26,000 participants in 5 countries (Haiti, India, Indonesia, Fiji, and Papua New Guinea). A 21 CFR Part 11 compliant electronic data capture system allowed deidentified data to be entered directly into a hand-held tablet via a mobile data management solution (‘App’) called CliniTrial (CliniOps, Fremont, CA). Data were synchronized regularly over the internet from all study sites through a secured Amazon Virtual Private Cloud server and compiled into one complete dataset. Validation checks and automated alert checks were programmed into the electronic data capture system to maintain a high level of data quality at the points of entry. The current study included participants that met the inclusion/exclusion criteria and received treatment (IDA or DA) in the LF clinical trial [6], and were ≥ 90 cm in height. Age, sex, country, height and weight were the primary variables of interest in the present study.

### Statistical methods

**Fig 1:**
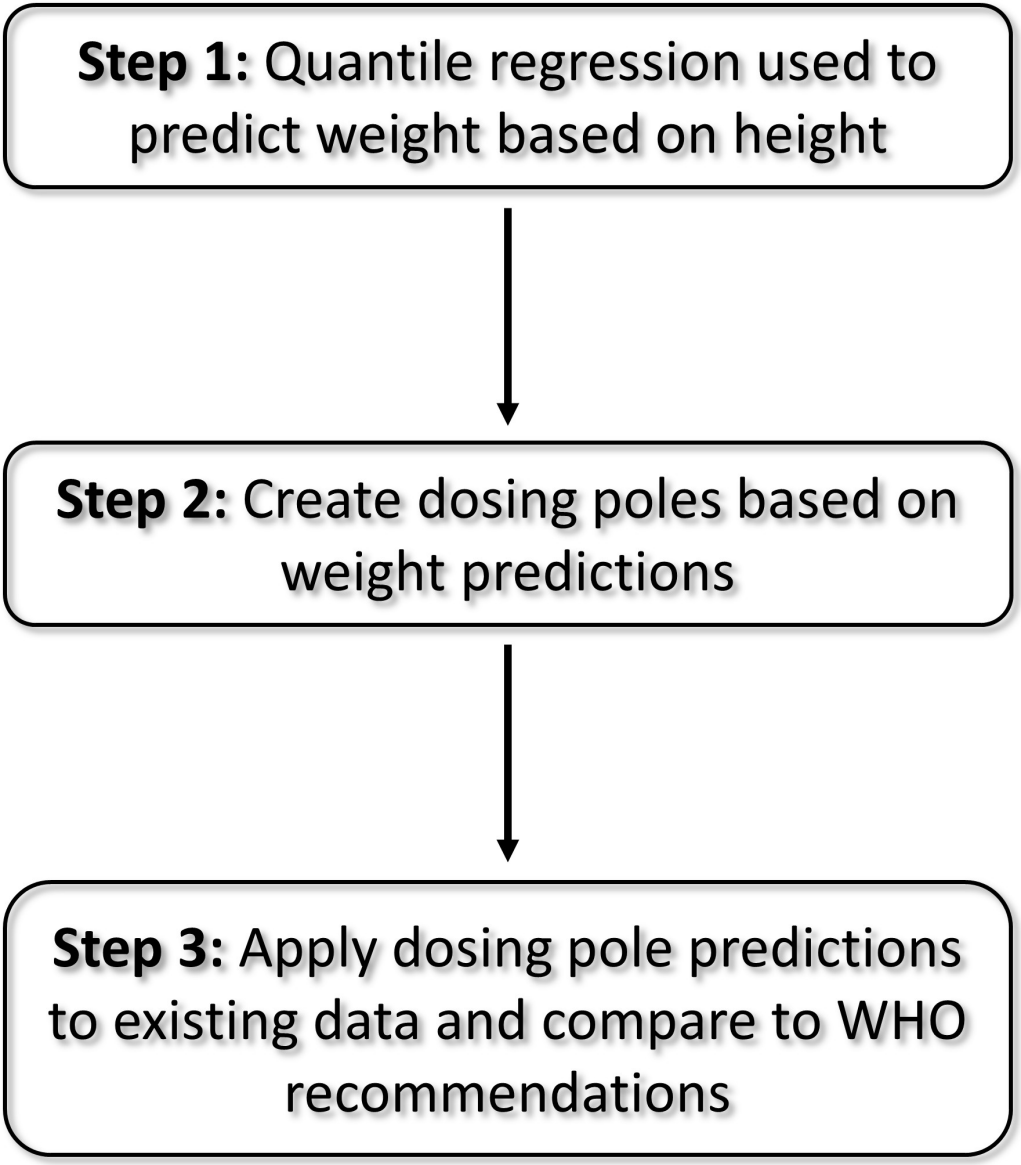
3-step modeling process.

Our modeling process involved three steps (Fig 1). The first step was to use quantile regression to predict weight based on height. Because of the myriad factors that influence weight as a person ages, weight and height become increasingly decoupled as individuals age. Quantile regression is a statistical approach that enables the data to be modeled across a range of quantiles [11, 12]. Quantiles correspond to the proportion of observations below a threshold. For example, in the context of quantile regression, the 0.25 quantile corresponds to the point at which ~25% of the observations fall below the regression line and ~75% of the observations are above the regression line. For our height-weight quantile regressions, the estimates for lower quantiles emphasize the prediction of lighter individuals and estimates for higher quantiles emphasize predictions towards heavier individuals. This approach allowed us to create and evaluate many different dosing pole scenarios (“dosing poles”) and choose the pole(s) that best meets the study objectives. In our quantile-regression models we employed a range of quantiles (0.10 to 0.90 at intervals of 0.05 for a total of 17 quantiles) to ensure that we captured a breadth of relationships between height and weight. In addition to Global models (no stratification of data, 1 model for each of the 17 quantiles), we performed analyses for multiple strata to determine whether predictions improved when data were stratified by Country (5 countries x 17 quantiles), Sex (2 sexes x 17 quantiles), or Country x Sex (5 countries x 2 sexes x 17 quantiles), resulting in a total of 306 quantile-regression models. Weight outcomes were log transformed prior to analysis and model predictions were then back-transformed (antilog [model prediction]) to obtain predicted weights on the original scale of the data. This allowed us to capture the nonlinear association between height and weight. Likelihood-ratio test statistics were used to obtain *P*-values. *P*-values < 0.05 were considered significant. All quantile-regression analyses were conducted using PROC QUANTREG in SAS version 9.4 (SAS Institute Inc., Cary, NC).

The second step was to use predicted weights from our step 1 models to create height-interval dosing poles. Our “gold standard” (hereafter recommended dosage) for weight-based dosing is based on the WHO GPELF recommendations and modified from the LF drug-safety clinical trial [6] so that the weight midpoints for the different dosages provided 6 mg/kg for DEC and 200 μg/kg for IVM (Table 1). The quantile-regression weight predictions were converted into tablet numbers using the weight ranges in Table 1. The predicted number of tablets for a given height were then operationalized as “full-dose” DOLF dosing poles.

**Table 1:** Weight based dosage modified from the global mass drug administration safety study.

Drug	Weight range (kg)	# tablets (mg)
**DEC**^#^	15–25	1 (100 mg)
	26–41	2 (200 mg)
	42–58	3 (300 mg)
	59–75	4 (400 mg)
	76–92	5 (500 mg)
	≥ 93	6 (600 mg)
IVM*	15–22	1 (3 mg)
	23–37	2 (6 mg)
	38–52	3 (9 mg)
	53–67	4 (12 mg)
	68–82	5 (15 mg)
	83–97	6 (18 mg)
	≥ 98	7 (21 mg)

^#^: DEC = Diethylcarbamazine; note that in India age-based dosing was used during the clinical trial.

*: IVM = Ivermectin

The third step was to assess how well the DOLF dosing pole predictions (step 2), the WHO age-based DEC dosing [9], and the WHO height-based (dosing pole) IVM dosing [8] corresponded to the recommended weight-based dosing (Table 1) using the observed weights from the LF clinical trial dataset. We also created two “hybrid” dosing poles that combine criteria from the WHO IVM dosing pole and the full-dose DOLF (IVM) dosing pole providing in a single pole that can be used for both IVM and DEC dosing. The hybrid poles are identical to the full-dose DOLF IVM dosing poles, except the hybrid poles included a maximum number of 4 (Hybrid 4) or 6 (Hybrid 6) tablets, and all participants ≥ 90 cm receive at least 1 tablet (participants < 90 cm were excluded from the dataset prior to analysis). Using a single pole for DEC and IVM and administering a small number of tablets that constitutes an adequate dose is desirable from both an implementation standpoint (individuals may be more likely to adhere to treatment with fewer tablets and MDA is simplified by use of a single dosing pole) and from a cost perspective. If the hybrid dosing poles perform similarly to the full-dose poles, then the hybrid poles would be the preferred option. The different dosing options (WHO, and DOLF full-dose and hybrid poles) were assessed by estimating the percentage of subjects that would have received below (BRD), above (ARD), or the recommended weight-based dosage.

## Results

### Cohort characteristics

Height and weight data from 26,821 individuals were included in this analysis. Sex of participants was balanced within and among countries with the percentage of males ranging from 47% to 53%. There was a 12-year range in median age, with Haiti having the youngest participants (age: median [IQR] = 18 [11, 30] years; 51.6% adults) and Fiji having the oldest participants (age: median [IQR] = 30 [12, 49] years; 62% adults). There was substantial variability in height and weight across the different countries. Participants in Indonesia had the lowest mean height (144.5 cm) and weight (38.7 kg), and participants in Fiji had the greatest mean height (159.8 cm) and weight (69.5 kg) (Table 2). Histograms that provide a graphical summary of the distributions for the variables in Table 1 are included as a supplement (S1 Fig).

**Table 2:** Descriptive statistics stratified by country.

Characteristic	Group	Fiji	Haiti	India	Indonesia	PNG
Sex	Female	1629 (47.5%)	3189 (53.2%)	4575 (51.4%)	1990 (50.7%)	2147 (47.1%)
	Male	1801 (52.5%)	2808 (46.8%)	4331 (48.6%)	1935 (49.3%)	2416 (52.9%)
Age (yrs)	NA	30 (12, 49)	18 (11, 30)	23.5 (13, 36)	20 (11, 39)	22 (14, 36)
Age group	Adult (≥18)	2127 (62%)	3096 (51.6%)	5630 (63.2%)	2184 (55.6%)	2823 (61.9%)
	Child (<18)	1303 (38%)	2901 (48.4%)	3276 (36.8%)	1741 (44.4%)	1740 (38.1%)
Height (cm)	NA	159.8 ±± 18.3	152.7 ± 19.5	146.6 ± 17.1	144.5 ± 18.4	148.5 ± 15
BMI (kg/m^2^)	NA	26 ± 7.9	20.9 ± 5.6	19.5 ± 4.6	17.8 ± 3.3	21.1 ± 3.8
Weight (kg)	NA	69.5 ± 28.4	51.1 ± 21.1	43.4 ± 15.5	38.7 ± 13.7	47.8 ± 14.4

Estimates reported as n (%), mean ± SD, or median (Q1, Q3).

### Comparisons of weight-based dosing to the WHO recommended IVM dosing by height and DEC dosing by age

**Fig 2:**
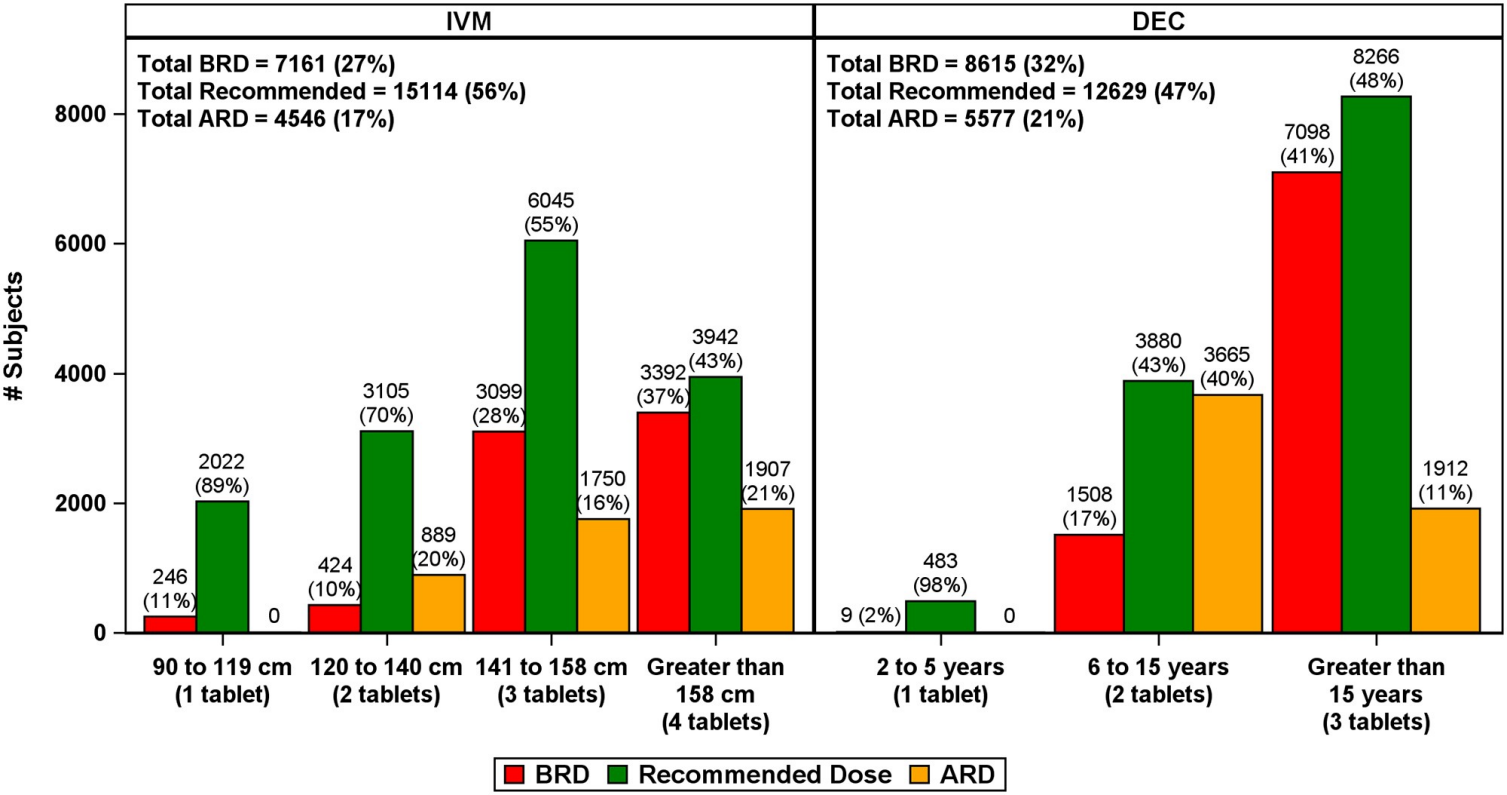
WHO age-based (DEC) and height-based (IVM) dosing compared to recommended weight-based dosing. Percentages calculated based on the number of participants equal to the recommended dose (recommended), above the recommended dosage (ARD), or below the recommended dose (BRD). Values reported above bars correspond to the n (%) within the dosage group, and values in the inset correspond to the overall n (%).

Very different dosing recommendations were obtained with different dosing algorithms (Fig 2). The WHO dosing pole for IVM agreed with the weight-based dosing in about 56% of participants; it resulted in below the recommended dosage (BRD) for 27% of the participants and above the recommended dosage (ARD) for 17% of the participants. Both BRD and ARD were more frequent in people with heights > 140 cm. Dosing discrepancies were more frequent with age-based dosing for DEC; it agreed with weight-based dosing in 47% of participants, with ARD and BRD rates of 21% and 32%, respectively. The percentage of participants receiving ARD was greatest for the 6 to 15-year age group and the percentage receiving BRD was greatest for people older than 15 years. When the analysis was restricted to adult (≥ 18 years) males, BRD percentages were 39% for IVM and 54% for DEC.

### Step 1: Height-weight quantile regression results

**Fig 3:**
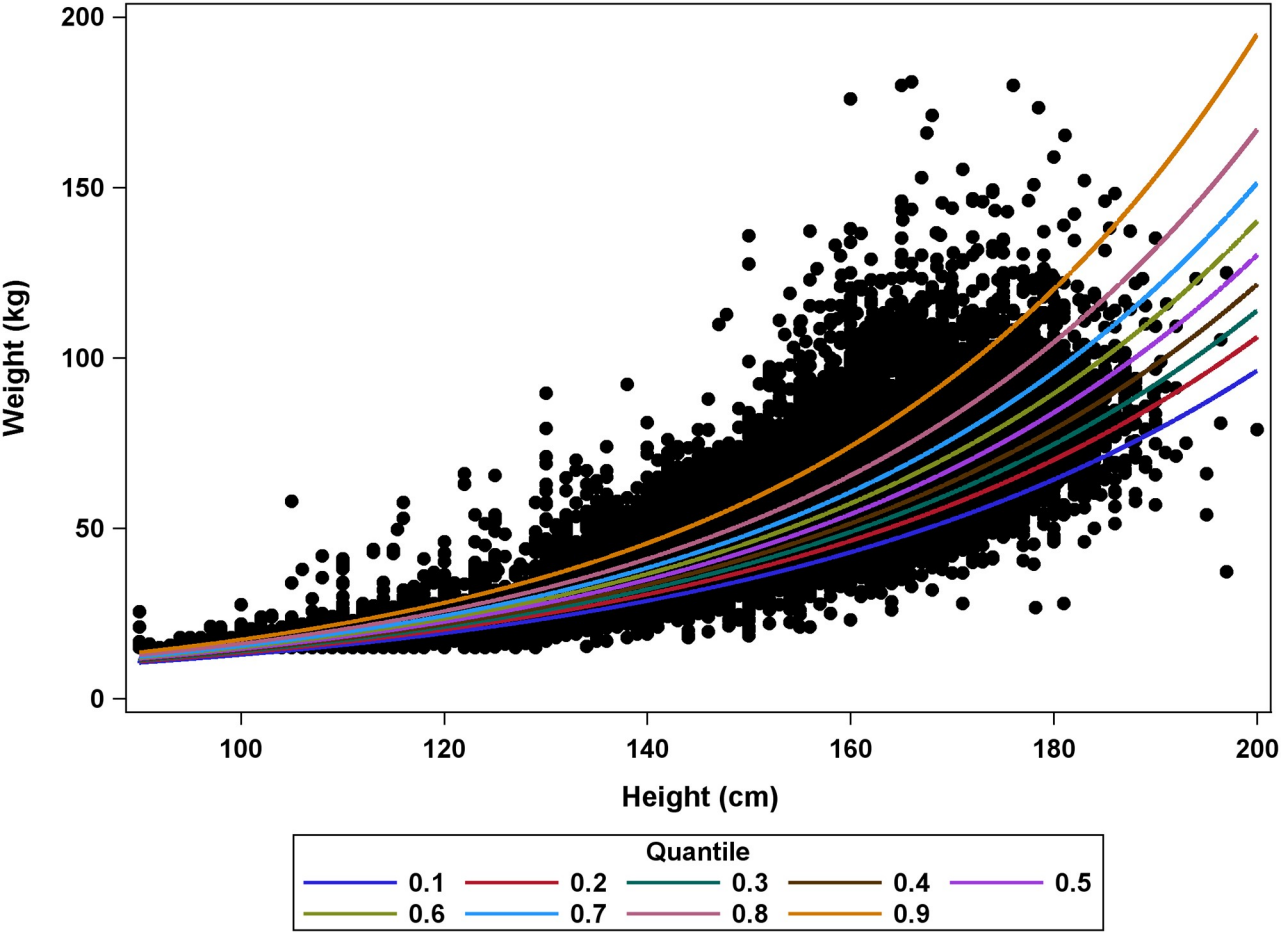
Quantile regression predictions of weight from height for Global models.

The quantile regression analyses revealed substantial heterogeneity in model predictions across quantiles. For all 306 quantile-regression models, the slope estimates showed significant positive associations between height and weight, regardless of quantile. Slope estimates and/or intercept estimates generally increased with quantile indicating a greater predicted weight for a given height in higher quantiles (S1 Table). Plots of the model predictions from the Global models (without stratification by country, sex, or age) showed that differences between model predictions increased with height, with the largest differences for the 10^th^ and 90^th^ quantile models for taller participants (Fig 3). For example, at a height of 95 cm the model predictions were relatively close for the 10^th^ and 90^th^ quantiles with predicted weights of 11.6 kg and 15.3 kg, respectively. However, at a height of 180 cm, the 10^th^ quantile model predicted weight of 64.7 kg, while the 90^th^ quantile model predicted a weight of 119.8 kg.

**Fig 4:**
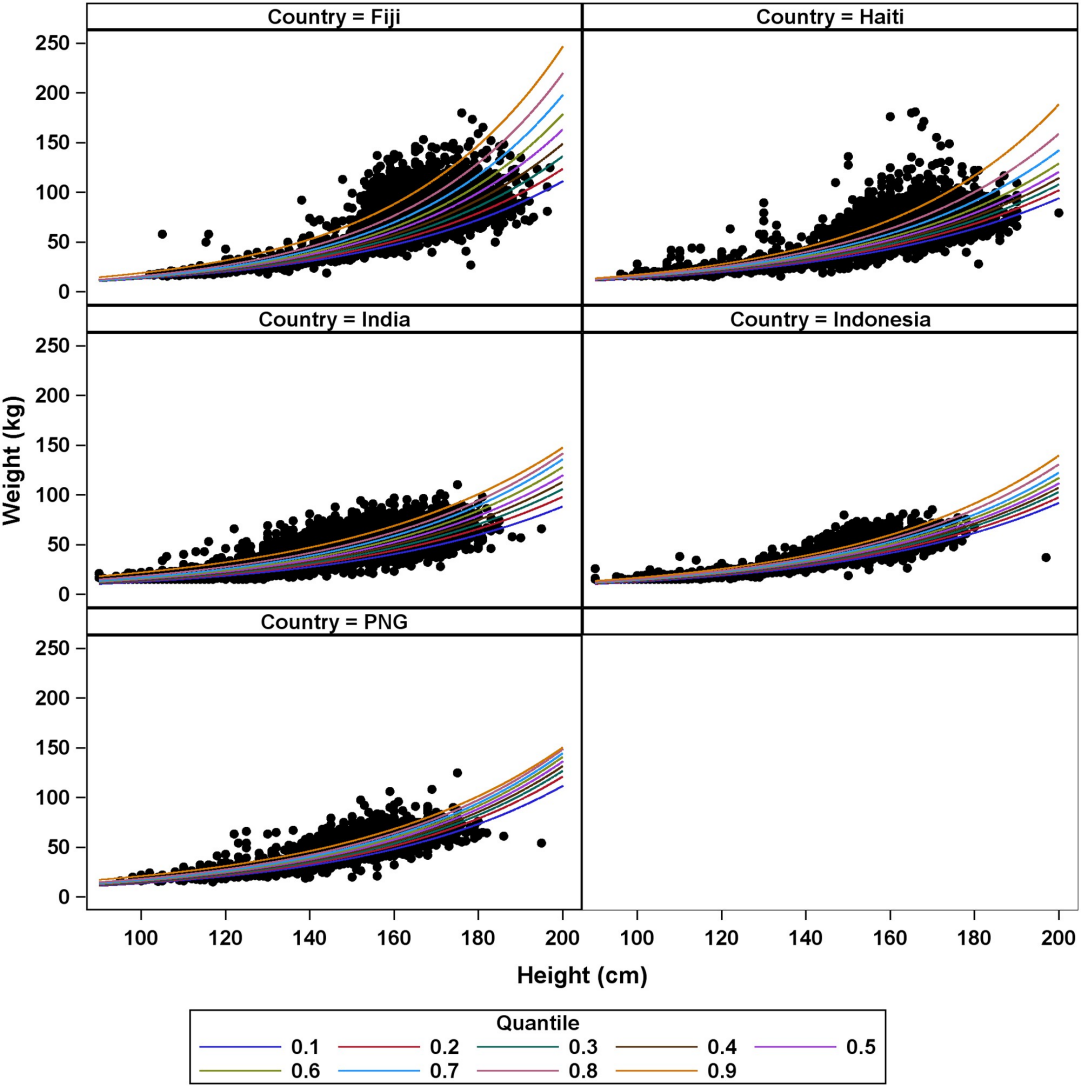
Quantile regression predictions for models stratified by country.

The country-specific models revealed marked variability in height-weight relationships between countries. Fiji and Haiti generally had the highest predicted weights for a given height, and Indonesia had the lowest, with India and PNG falling between these extremes (Fig 4). Weight prediction differences between countries increased both with subject height as well as quantile. Country-specific height-weight slope estimates indicated that for all quantiles Fiji weight predictions had the largest increase in predicted weight with height (S2 Fig).

**Fig 5:**
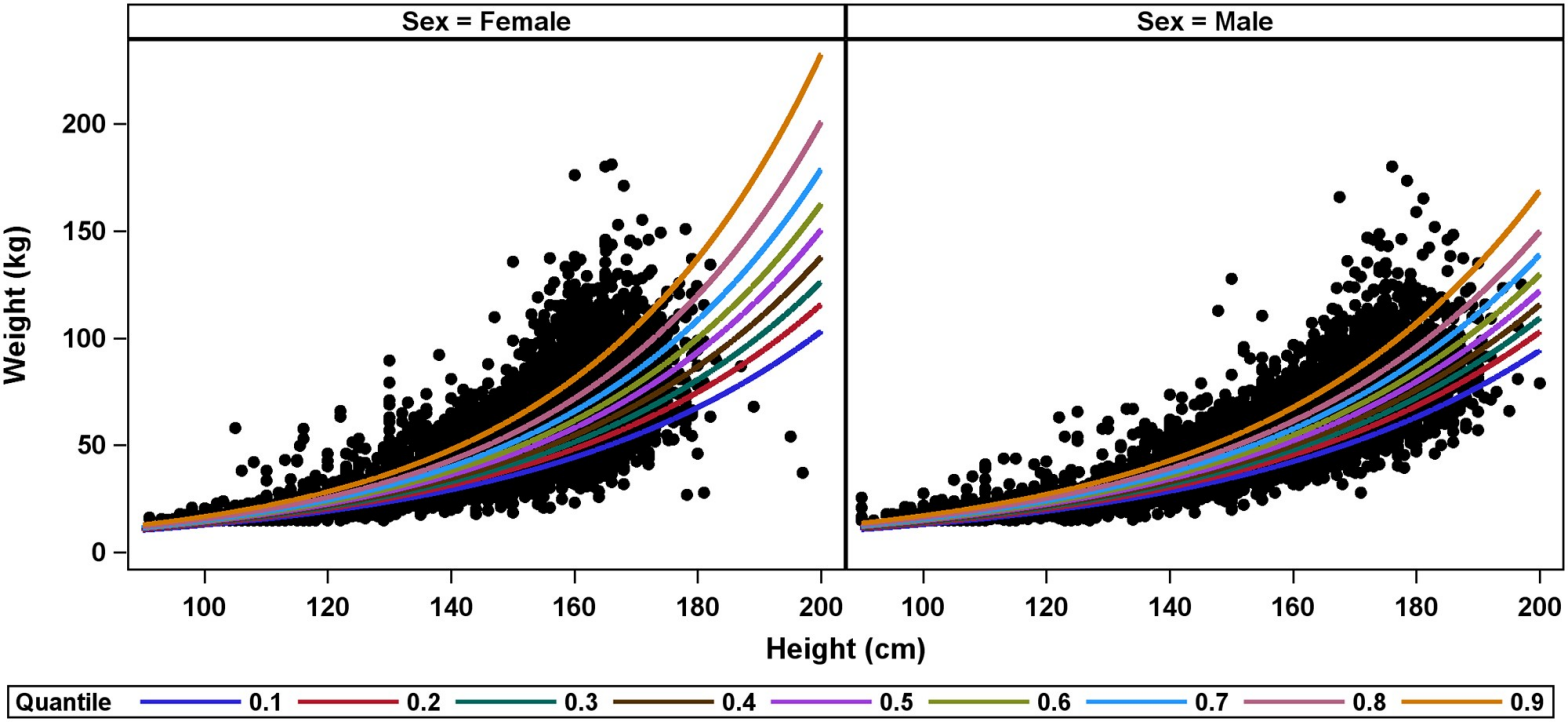
Quantile regression predictions for models stratified by sex.

When we stratified the data by sex, we found that females were heavier for a given height than males, as reflected in both the parameter estimates and the model predictions. Similar to the other strata, the differences between the quantile-regression predictions increased with height, and the predicted weight differences by sex were greater in larger quantiles (Fig 5). Sex-specific slope estimates indicated that females had steeper slopes than males for all quantiles (S3 Fig). Models stratified by both country and sex revealed some variability between sexes within a country, but generally the difference was small relative to inter-country differences (S1 Table).

### Step 2: Dosing poles based on model predictions

**Fig 6:**
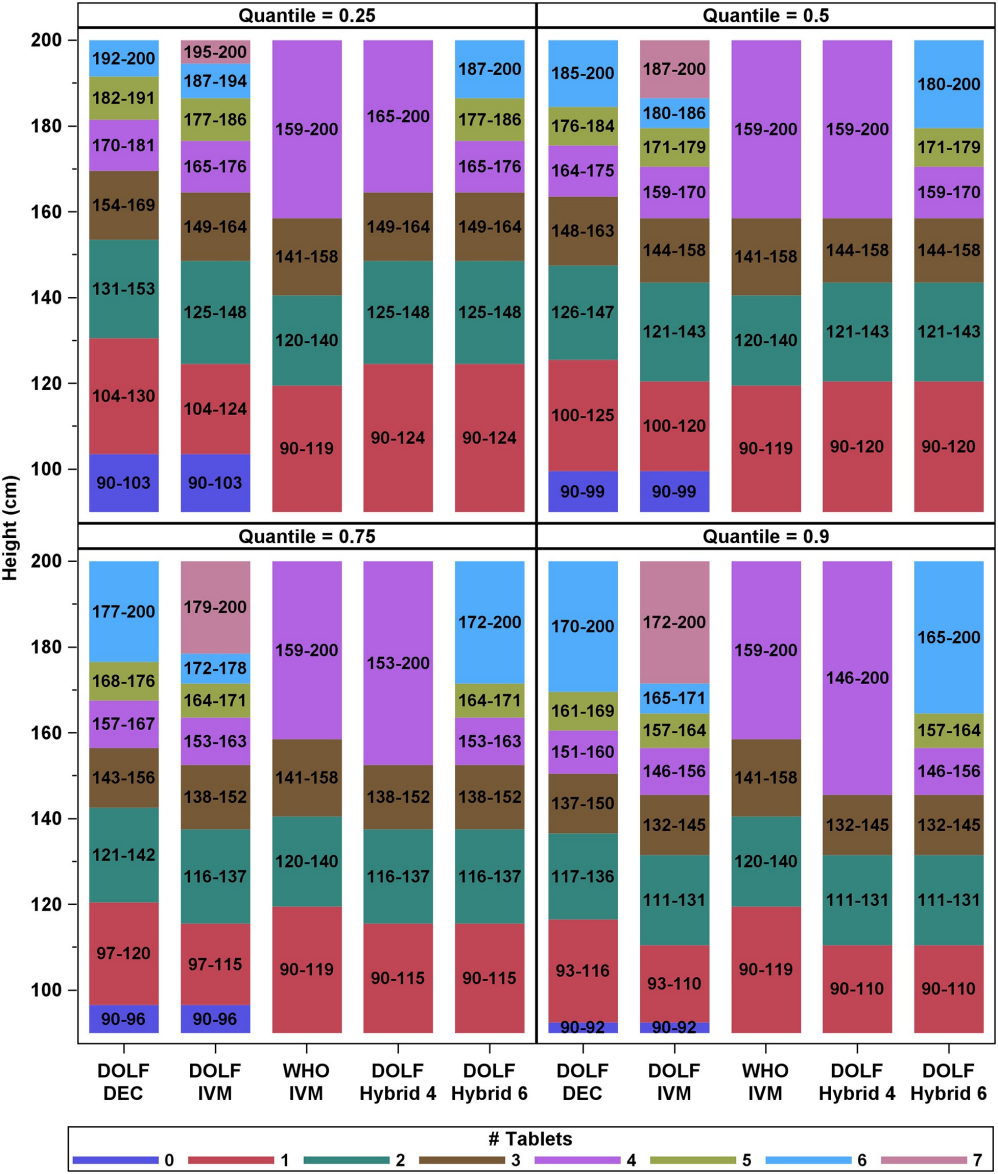
Dosing poles for Global models. Different colors correspond to different # of tablets administered, and numbers in bars correspond to the height range (in cm) for each tablet group. Hybrid 4 corresponds to the 4-tablet maximum dosing pole, and Hybrid 6 corresponds to the 6-tablet maximum dosing pole. The hybrid poles can be used for dosing either IVM or DEC.

Results from our weight predictions were used to make dosing poles based on observed subject heights. Compared to the WHO height-based IVM dosing pole and the WHO age-based DEC dosing, the “full dose” DOLF dosing poles had a greater range of dosages (0–6 tablets for DEC and 0–7 tablets for IVM). Results from our Global models (combined data from all countries) revealed that for both DEC and IVM, the minimum height required for a subject to receive any specific number of tablets was consistently lower for the higher quantile models (Fig 6). For example, at the 25^th^ quantile the minimum height to receive a single tablet of IVM was 104 cm, whereas for the 90^th^ quantile the minimum height to receive a single tablet of IVM was only 93 cm. For the WHO IVM dosing pole, the minimum height required to receive a specific dosage (≤ 4 tablets) was lower than the DOLF models for smaller quantiles (0.25 and 0.50) and higher for larger quantiles (0.75 and 0.90) except for “one-tablet”. Results from the hybrid poles that can be used for dosing DEC or IVM with either a 4-tablet maximum (Hybrid 4) or a 6-tablet maximum (Hybrid 6) showed that, in contrast to the full-dose poles, all participants 90 cm or greater would receive at least one tablet. Furthermore, for the highest quantile model (0.90) there was a much wider range of participants that would receive four tablets or more (147–200 cm) as compared to the WHO IVM pole (159–200). Dosing poles varied somewhat according to strata, with the most dramatic changes attributed to Fiji, which was the country with the lowest tablet thresholds of any of the dosing poles (S4 Fig).

### Step 3: Application of dosing pole models to the DOLF safety study dataset with comparisons to weight-based and WHO height and age-based dosing

**Fig 7:**
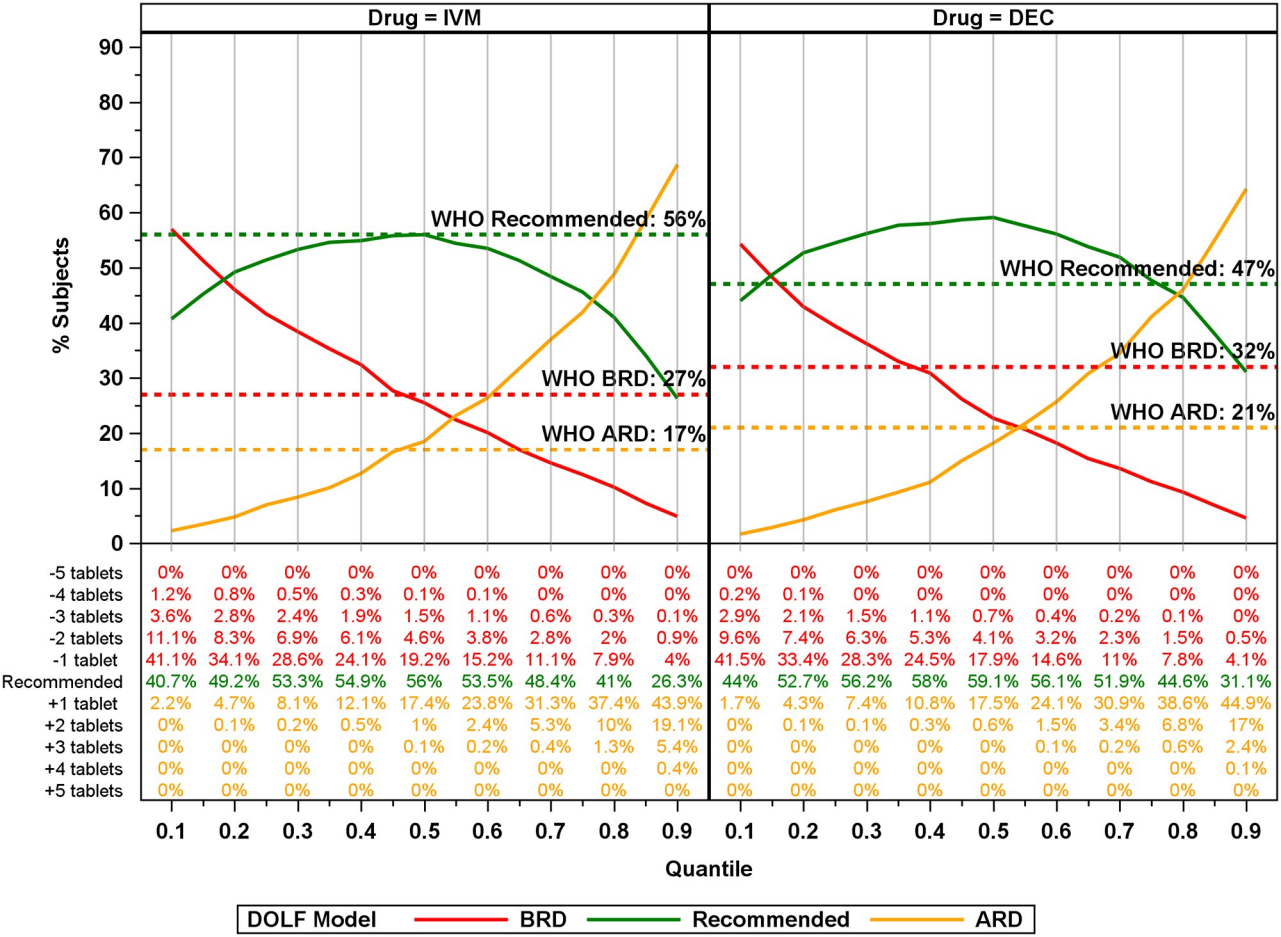
Global model plots (6 [DEC] and 7 [IVM] tablet maximum) of % BRD, ARD, and at recommended dosages for IVM and DEC for all quantiles. Colored lines correspond to the dosage category with the recommended dosage colored green, below the recommended dosage (BRD) colored red, and above the recommended dosage (ARD) colored orange. Solid lines correspond to model predictions for the percentage of participants receiving ARD, BRD or the recommended dosage for the model. Horizontal lines correspond to WHO recommended dosages. Numbers below the plot correspond to percentages of total participants for the number of tablets ARD, BRD, or recommended.

Application of the model-based dosing poles to the DOLF dataset revealed a marked improvement in dosing compared to the current WHO recommendations. Using dosing poles from the stratified analyses (Country, Sex, Country x Sex) produced only marginal improvements compared to unstratified Global models with regards to dosing (See S5 Fig). Therefore, we decided to focus on results from the Global models here. For all models there was an inverse relationship between the percentage of participants above the recommended weight dosage (ARD) and below the recommended weight dosage (BRD): % BRD declined with quantile reaching a minimum at quantile 0.90 whereas the minimum % ARD was at quantile 0.10 (Fig 7). The Global DOLF models resulted in lower % BRD values for quantiles ≥ 0.50 compared to WHO height-based IVM dosing, and greater than quantile 0.35 compared to the WHO age-based DEC dosing. The percentage receiving recommended dosing for the DOLF IVM models were approximately equal to the percentage receiving recommended dosing for the WHO-IVM dosing between quantiles 0.3 and 0.6. For DEC, the percentage receiving recommended dosing for the DOLF model exceeded the percentage receiving recommended dosing for the WHO-DEC dose between quantiles 0.15 and 0.70. The dosing pole that minimized the percentage receiving BRD occurred at the 0.90 quantile, with 5% or less of participants BRD for both IVM and DEC resulting in 22% and 27% improvements over the existing WHO dosing methods, respectively. This dosing pole resulted in 69% (IVM) and 64% (DEC) participants ARD with only 5.8% of participants receiving more than two tablets ARD for IVM, and only 2.5% receiving more than two tablets ARD for DEC. Importantly, the 0.90 quantile DOLF dosing poles dramatically reduced BRD for adult males, with estimates of the percentage receiving BRD less than 3% for IVM and DEC compared to 39% and 54% underdosing for the WHO IVM height pole and WHO DEC age-based dosing, respectively.

**Fig 8:**
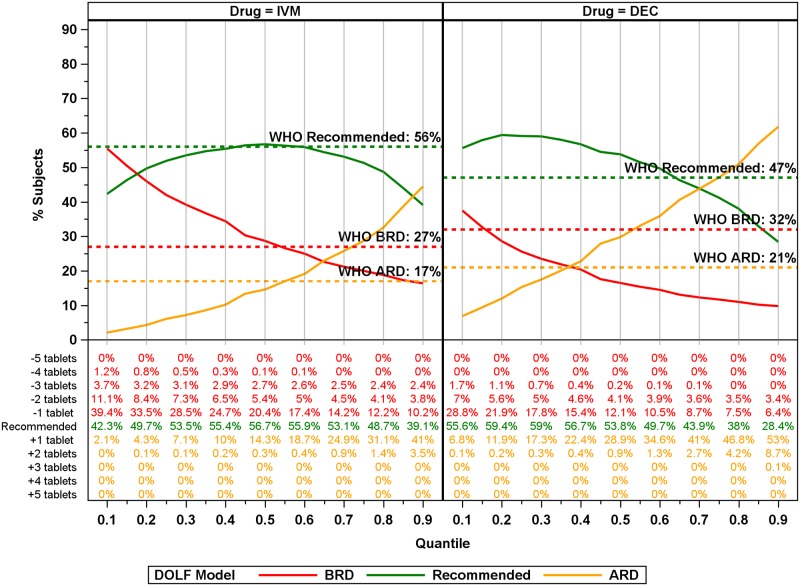
Global DOLF 4-tablet maximum hybrid model plots of the percentage receiving BRD, ARD, and recommended dosages for IVM and DEC for all quantiles. Colored lines correspond to the dosage category with the recommended dosage colored green, below the recommended dosage (BRD) colored red, and above the recommended dosage (ARD) colored orange. Solid lines correspond to model predictions for the % of participants ARD, BRD or at recommended dosage for the model. Horizontal lines correspond to WHO recommended dosages. Numbers below the plot correspond to percentages of total participants for the number of tablets ARD, BRD, or recommended.

The improvement (compared to WHO) in the percentage of participants receiving BRD for IVM with 4-tablet Global hybrid dosing poles was lower than that obtained with the full-dose DOLF IVM dosing pole. For DEC, the BRD percentage fell below the WHO DEC BRD at lower quantiles than the full-dose DOLF DEC dosing pole (Fig 8). The DEC 4-tablet hybrid pole performed better than the full-tablet pole (with respect to BRD) for many of the quantiles because we applied the DOLF IVM dosing pole to DEC which has lower weight thresholds for different dosing levels (see Table 1 and Fig 6). For IVM the hybrid 0.90 quantile dosing pole had 11% lower BRD compared to the WHO IVM pole, and for DEC the 0.90 quantile dosing pole had 22% lower BRD compared to the WHO age-based dosing.

**Fig 9:**
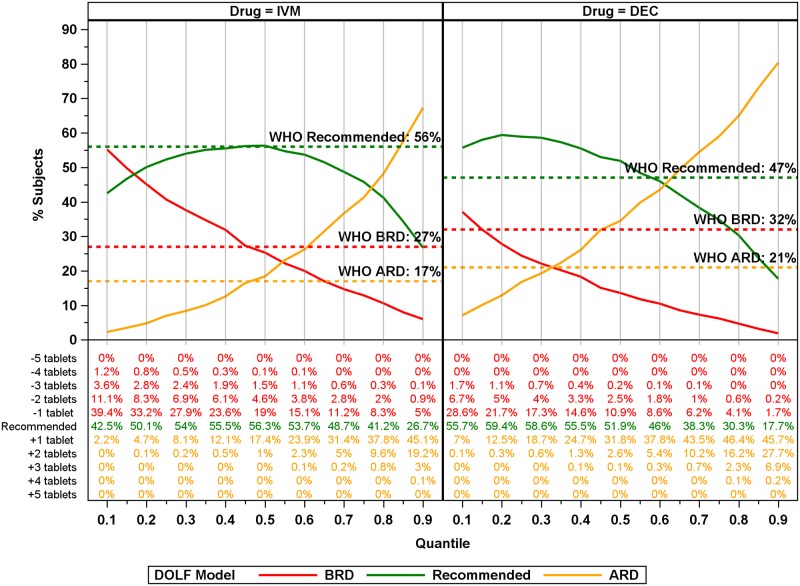
Global DOLF 6-tablet maximum hybrid model plots of % BRD, ARD, and recommended dosages for IVM and DEC for all quantiles. Colored lines correspond to the dosage category with the recommended dosage colored green, below the recommended dosage (BRD) colored red, and above the recommended dosage (ARD) colored orange. Solid lines correspond to model predictions for the percentage of participants ARD, BRD or at recommended dosage for the model. Horizontal lines correspond to WHO recommended dosages. Numbers below the plot correspond to percentages of total participants for the number of tablets ARD, BRD, or recommended.

Similar to the 4-tablet hybrid pole, the 6-tablet hybrid percentage receiving BRD for DEC fell below the WHO BRD at small quantiles, and the percentage BRD was the lowest of any of the dosing poles based on Global models—reaching as low as 1.9% (Fig 9). The trade-off is that for the 0.90 quantile dosing pole, 80% of individuals were ARD for DEC with about 7.1% receiving three or more tablets above the recommended dosage. For IVM, the 6-tablet hybrid dosing pole at the 0.9 quantile performed similarly to the full-dose IVM pole with 6% of the participants BRD, 27% recommended, and 67% ARD.

**Fig 10:**
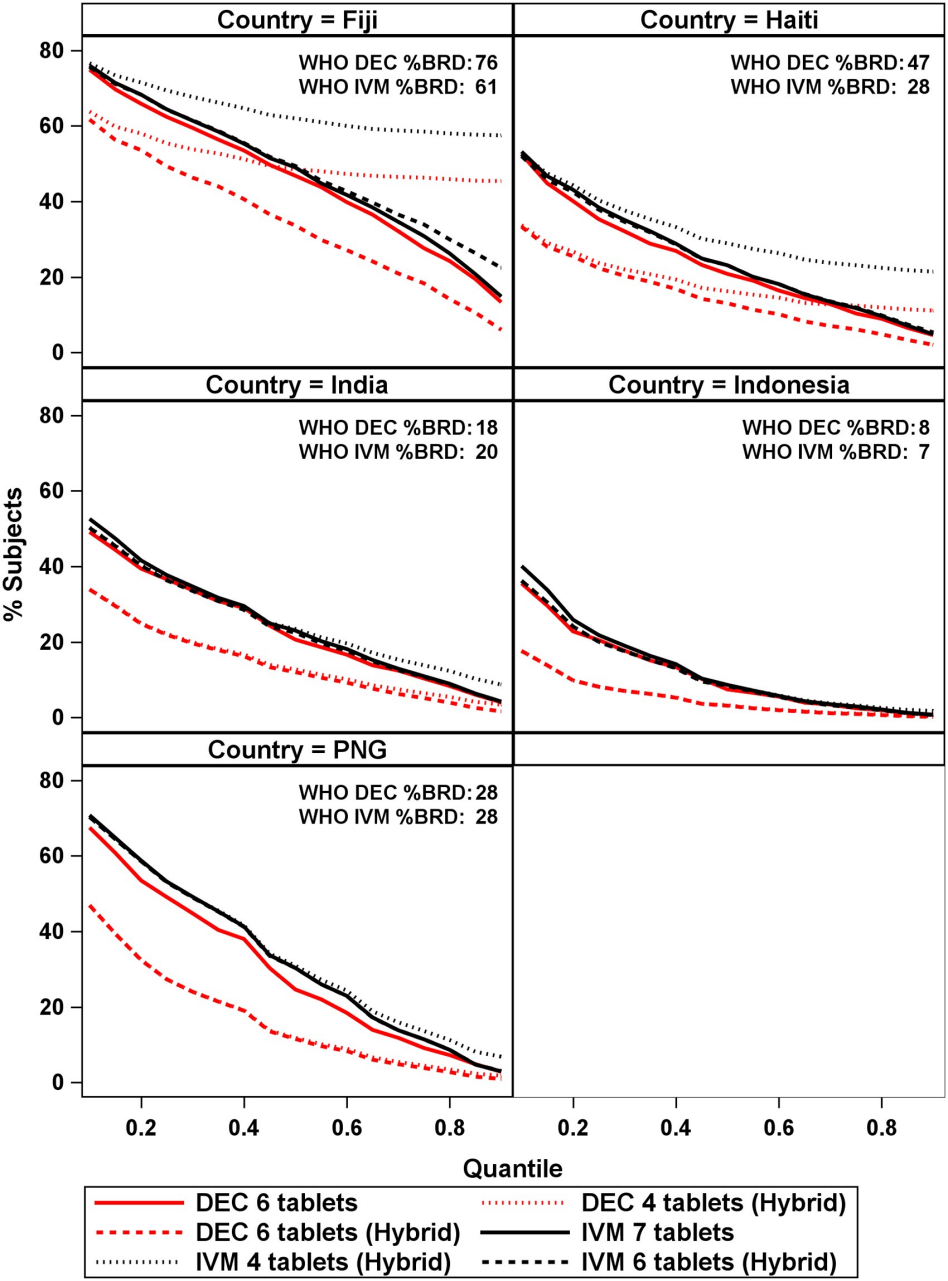
Percentage of participants below recommended dosage (BRD) across quantiles by country for the Global models.

Estimates of the percentage of participants BRD from the Global dosing poles applied to specific countries indicated that at larger quantiles the DOLF dosing poles generally improved upon the current WHO age and height-based dosing (Fig 10, inset and lines). In India, Indonesia, and PNG the 4 and 6-tablet Global models % BRD estimates were approximately equal to the full-dose dosing poles for IVM. However, both hybrid poles resulted in lower estimates of the percentage receiving BRD as compared to the full-dose poles for DEC over all quantiles. In Haiti, the 4-tablet hybrid dosing pole had poorer performance for IVM than the full-dose and 6-tablet hybrid dosing poles for most quantiles, and the 4-tablet hybrid pole performed better than the full-dose DEC poles in all but the most extreme (>0.70) quantiles. The 6-tablet hybrid pole outperformed the 4-tablet and full-dose poles for DEC across all quantiles in Haiti. In Fiji, the full-dose poles for DEC and IVM performed much better than the 4-tablet hybrid poles, especially for larger quantiles. The 6-tablet hybrid poles outperformed the full-dose and 4-tablet hybrid poles for DEC, and the estimates of the percentage receiving BRD for the 6-tablet hybrid pole were similar to the full-dose poles for IVM.

### Use of the WHO IVM pole for DEC compared to dosing based on age and the DOLF hybrid poles

We assessed whether use of the current WHO IVM dosing pole would have improved DEC dosing compared to age-based dosing. Our results indicated that the current WHO IVM pole would have resulted in 17% fewer participants receiving BRD compared to age-based dosing (15% vs 32%, Table 3). The consequence for that improvement was a 12% increase in ARD (33% vs 21%, Table 3). However, the WHO IVM pole did not perform as well as the Hybrid 4 and the Hybrid 6 DOLF dosing poles (0.90 quantile models) which had 22% and 30% fewer participants receiving BRD than age-based dosing, respectively. As indicated previously for IVM, the Hybrid 4 and Hybrid 6 poles reduce the percentage of participants receiving BRD compared to the WHO IVM pole by 11% and 21%, respectively (Table 3).

**Table 3:** Recommended, BRD, and ARD for WHO and DOLF hybrid dosing poles for DEC and IVM.

Drug	Group	WHO Age-Based	WHO IVM Pole	DOLF Hybrid 4	DOLF Hybrid 6
DEC	BRD	8615 (32%)	4103 (15%)	2620 (10%)	515 (2%)
	Recommended	12629 (47%)	13869 (52%)	7622 (28%)	4754 (18%)
	ARD	5577 (21%)	8849 (33%)	16579 (62%)	21552 (80%)
IVM	BRD	NA	7161 (27%)	4406 (16%)	1612 (6%)
	Recommended	NA	15114 (56%)	10477 (39%)	7152 (27%)
	ARD	NA	4546 (17%)	11938 (45%)	18057 (67%)

Hybrid dosing poles based on 0.90 quantile models.

## Discussion

Our study considered data from more than 26,000 participants, and we believe this is the largest single multi-country study to date that has evaluated different dosing pole recommendations for treating a neglected tropical disease (NTD). The results suggest that the current WHO age (DEC) and height-based (IVM) dosing recommendations for treatment of LF result in an excessive number of individuals receiving lower than weight-based recommended dosing. Importantly, we found that BRD was more frequent for adult males. Adult males are of particular concern for LF elimination programs because they often have higher LF infection rates than other demographic groups [13]. Through a 3-step modeling process we were able to develop dosing poles that have the potential to substantially reduce BRD, a reduction whose benefits far outweigh the downside of an increase in ARD. When we applied a single DOLF hybrid dosing pole (based on our Global models) with 4 or 6-tablet maximal dosing for both DEC and IVM, the percentage of participants with BRD were similar to those obtained with the DOLF full-dose models (6 tablet maximum for DEC [600 mg] and 7 tablet maximum for IVM [21 mg]), and similar to more complex models that would require separate dosing poles for different countries and sexes. Because IVM and DEC have large safety margins, we consider the BRD risk to outweigh the ARD risk. Therefore, we recommend the use of a single 6-tablet maximum hybrid dosing pole based on the Global model from the 0.9 quantile (Figs 6–9).

The dosing poles described in this study improve upon age or height-based dosing methods currently recommended by the WHO. Alexander et al. (1993) developed the current IVM dosing pole that is used by the WHO to treat LF based on weight data collected as part of an onchocerciasis clinical trial in Nigeria. This dosing pole has been widely applied in national MDA programs [14]. The data from our study suggest that improvements can be made to the WHO IVM dosing pole by lowering the height thresholds for dosing. That would result in a marked reduction in BRD (21% reduction for the 6-tablet hybrid pole). The age-based dosing approach for DEC is recommended by the WHO in their guidance manual for preventative chemotherapy [9], and it is also widely implemented as part of community MDA programs. Our results showed that age-based dosing would have resulted in 32% participants receiving BRD in this study, whereas the lowest level of BRD for the 6-tablet hybrid pole was 2% of participants. This discrepancy is largely driven by the low maximum dosage of 300 mg (3 tablets) for WHO age-based dosing compared to maximums of 600 mg (6-tablets) or 400 mg (4-tablets) for the DOLF height-based dosing models. Furthermore, we found that applying the existing WHO IVM dosing pole for DEC dosing would also lower BRD. Although the existing WHO IVM pole did not perform as well as the DOLF hybrid poles, this result indicates that substantial improvements in DEC dosing could be achieved with the legacy IVM dosing pole.

Providing MDA below weight-based recommended dosing may constrain the ability of community MDAs to interrupt disease transmission, which is a principal goal of the GPELF program. Based on estimates from our study, the WHO height-based dosing pole for IVM and the age-based dosing for DEC are likely to provide BRD to as high as one third of the populations in LF-endemic areas. Adult males are a cohort of particular concern because males typically have higher infection rates than females [13]. BRD percentages for this group using WHO age-based dosing were higher than the overall study cohort, reaching 54% of participants for DEC. Although the consequences of BRD on treatment efficacy and transmission are uncertain, the current WHO recommended dosing strategy might prolong the time required to interrupt LF transmission. The dosing pole-approach outlined in our study should reduce the percentage of individuals who receive BRD in community MDA programs where weight-based dosing is not feasible. Using our modeling approach, the trade-off in reducing BRD is that it increases ARD. Of the participants that received ARD in our study, most would have received 2 tablets or fewer above the weight-based recommended dosage (only 3% and 7% of total participants would have received more than 2 tablets above recommended dosage for IVM and DEC, respectively). However, we do not consider ARD to be a major concern, because there is evidence that IVM [15] and DEC [16] are safe and have relatively few side effects even at much higher doses than the weight-based doses recommended by the WHO. Safety margins and other considerations would need to be considered for development of dosing poles for different drug combinations.

Our 3-step modeling process is a novel approach for creating dosing poles that could be applied for mass distribution of other medications. Previous studies have described using height-based dosing poles for treatment of other NTDs [8, 17–19]. However, a variety of factors can affect height-weight relationships (e.g., age, sex, geographic location) which can lead to inaccurate dosing [20]. Thus, use of a single dosing pole may not be appropriate in all settings. In our study, we employed quantile regression which allows for many different dosing pole scenarios to be created and evaluated. Quantile regression has been used in a variety of fields such as econometrics [11, 21, 22], ecology and evolution [23–25], and medicine [26–28] to address questions that cannot readily be answered by standard analytic methods. To our knowledge quantile regression has not been employed previously to develop dosing poles. Data from our study indicate that there is a progressive decoupling of height and weight in larger and older individuals that limits the accuracy of using only height to predict weight. Quantile regression enabled us to “bias” our model predictions by quantile to allow our models to emphasize heavier individuals (to lower the percentage of participants receiving BRD) or to emphasize lighter individuals (to lower the percentage receiving ARD). The choice of whether to weight predictions towards a specific group for dosing purposes will depend on the goals and objectives of a particular program or study, and the safety margins of the medications used for MDA. In our study, the primary focus was to determine whether our models could reduce the amount of BRD that occurs when current WHO height- and age-based dosing recommendations are used for IVM and DEC, respectively. Therefore, we chose models that minimized percentage of participants receiving BRD (larger quantiles that weighted predictions towards heavier individuals). The 306 different statistical models developed in our study can be used to generate both full dose (7 for IVM and 6 for DEC) or hybrid (4 or 6 tablet maximum) dosing poles. These dosing pole models are available as a supplement (S1 Table) and could be employed in a variety of different contexts with guidance.

### Conclusions

Current WHO alternatives to weight-based dosing for mass treatment of LF are suboptimal. We have presented a modeling approach that offers an improved dosing method for administering IVM and DEC to LF-endemic populations. Our recommendation for mass treatment of LF is that a single 6-tablet maximum dosing pole from the 0.90 quantile should be used in all contexts. Areas with smaller individuals (e.g., in this study India and Indonesia) may be able to employ the 4-tablet dosing pole without appreciable increases underdosing. Results from our modeling effort go beyond recommending a single dosing pole solution for LF treatment. Users of our models can take into account a variety of competing goals and objectives to choose the dosing pole(s) that best corresponds to their setting. Improved dosing may enhance the efficacy of MDA and accelerate LF elimination.

## Supporting information

S1 Fig Histograms for demographic variables stratified by country. (PDF)

S2 Fig Estimates on the log scale of height-weight slope parameters and 95% CIs by quantile. (PDF)

S3 Fig Estimates on the log scale of height-weight slope parameters and 95% CIs by quantile stratified by sex. (PDF)

S4 Fig Dosing pole graphs with height cut offs for quantiles 0.25, 0.5, 0.75, and 0.9 for all of the stratified and hybrid models: Sex, country, and country x sex. (PDF)

S5 Fig Bar plots of percentages below, above, and at recommended weight-based dosage for each model. (PDF)

S1 Table Quantile regression parameter estimates stratified by model group and quantile. *P*-values correspond to a likelihood-ratio test that the slope coefficient = 0. All parameter estimates are presented on the log scale. Model predictions were obtained by taking the antilog of the model predictions.
(PDF)
