# Supplementary figures and images for: Dosing pole recommendations for lymphatic filariasis elimination: A height-weight quantile regression modeling approach
Source: PLoS Negl Trop Dis. 2019 Jul 17;13(7):e0007541. doi: 10.1371/journal.pntd.0007541 (PMC6663033; doi:10.1371/journal.pntd.0007541)

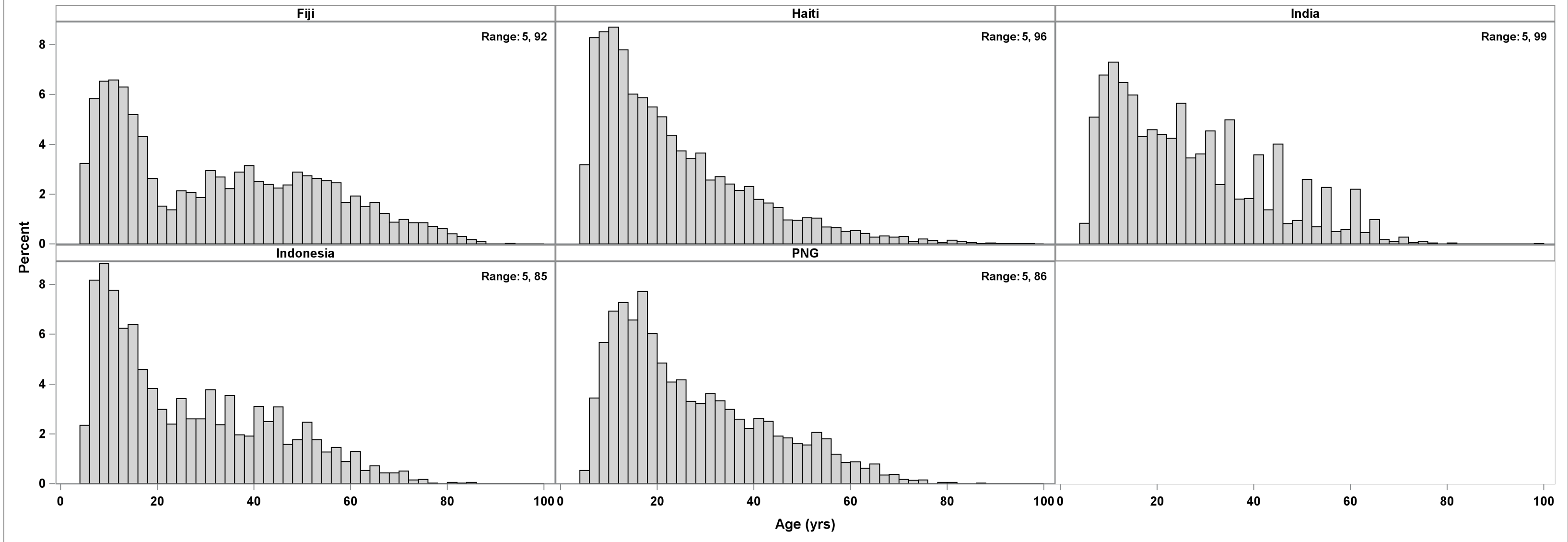

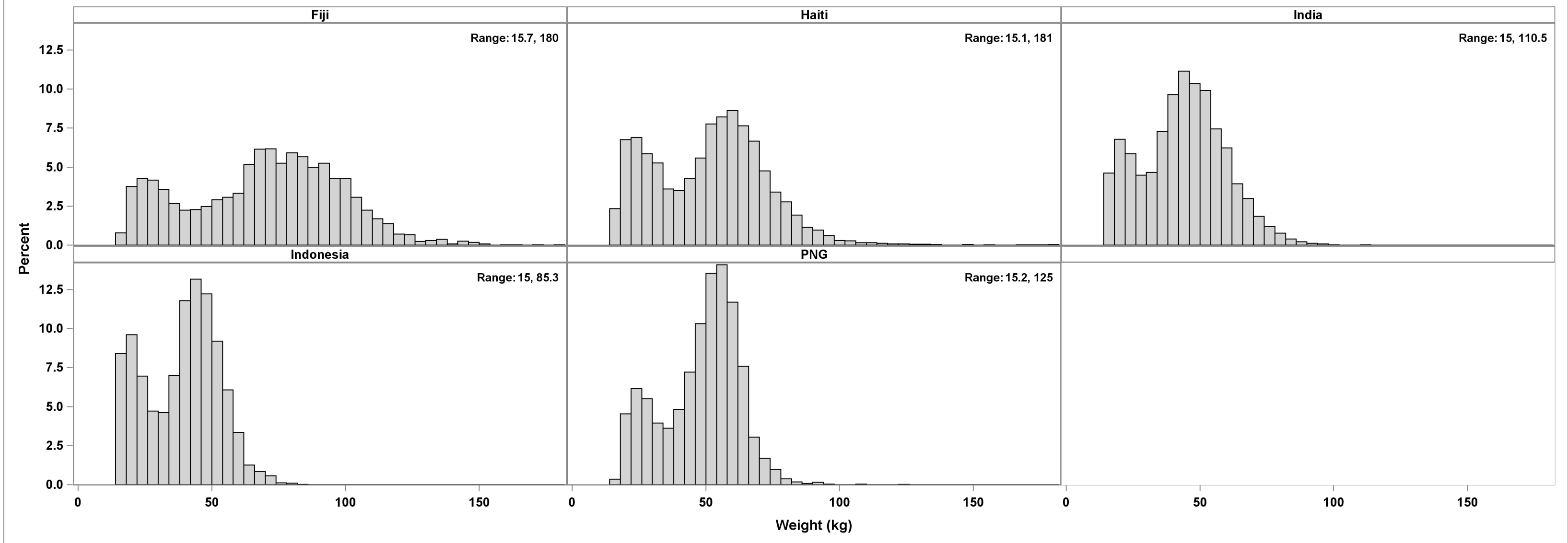

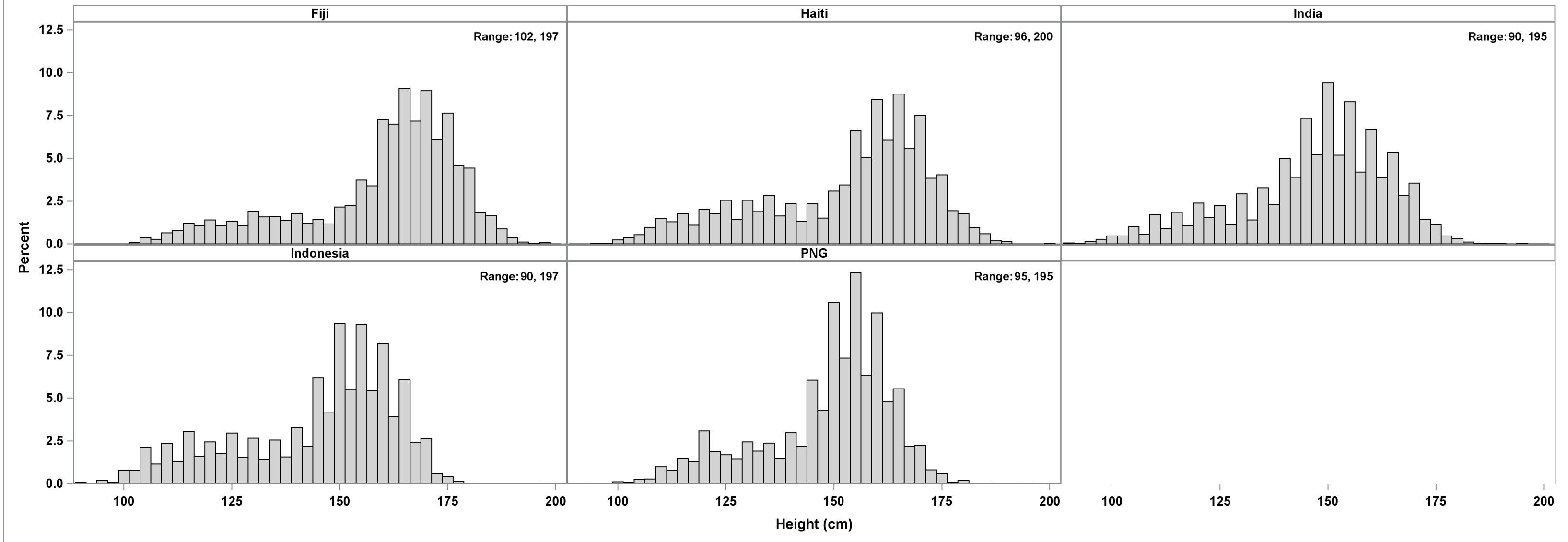

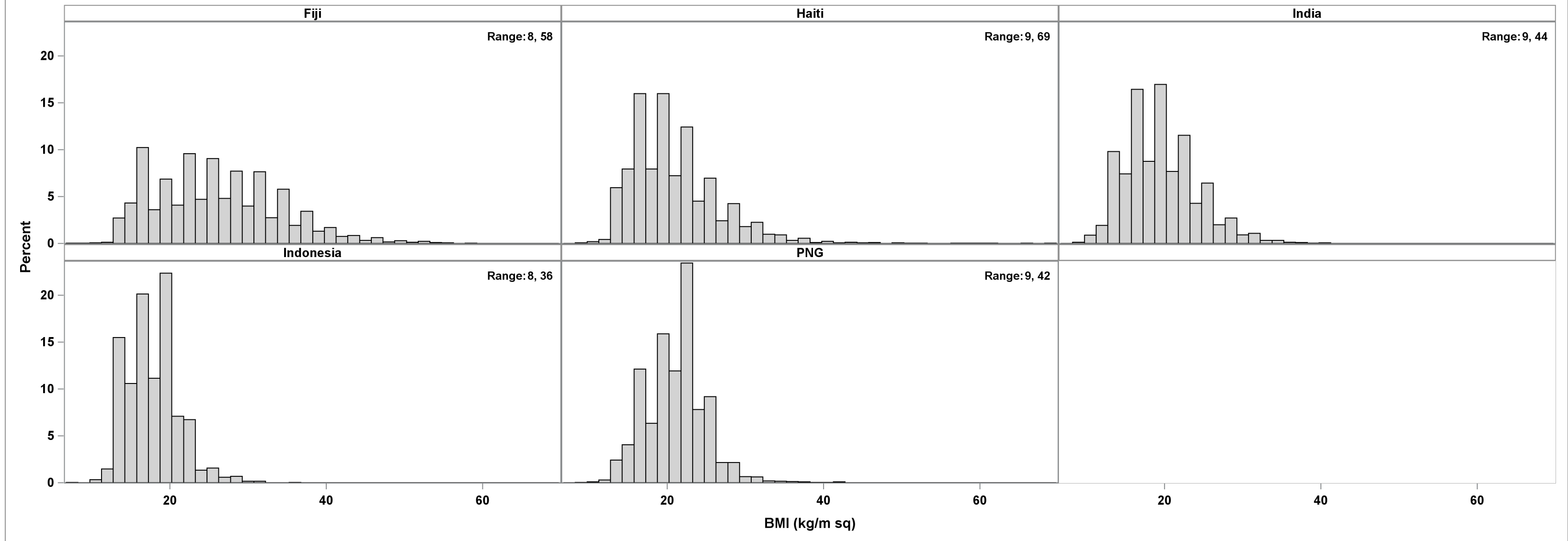

Supplement: S1 Fig — (PDF) [file pntd.0007541.s001.PDF]

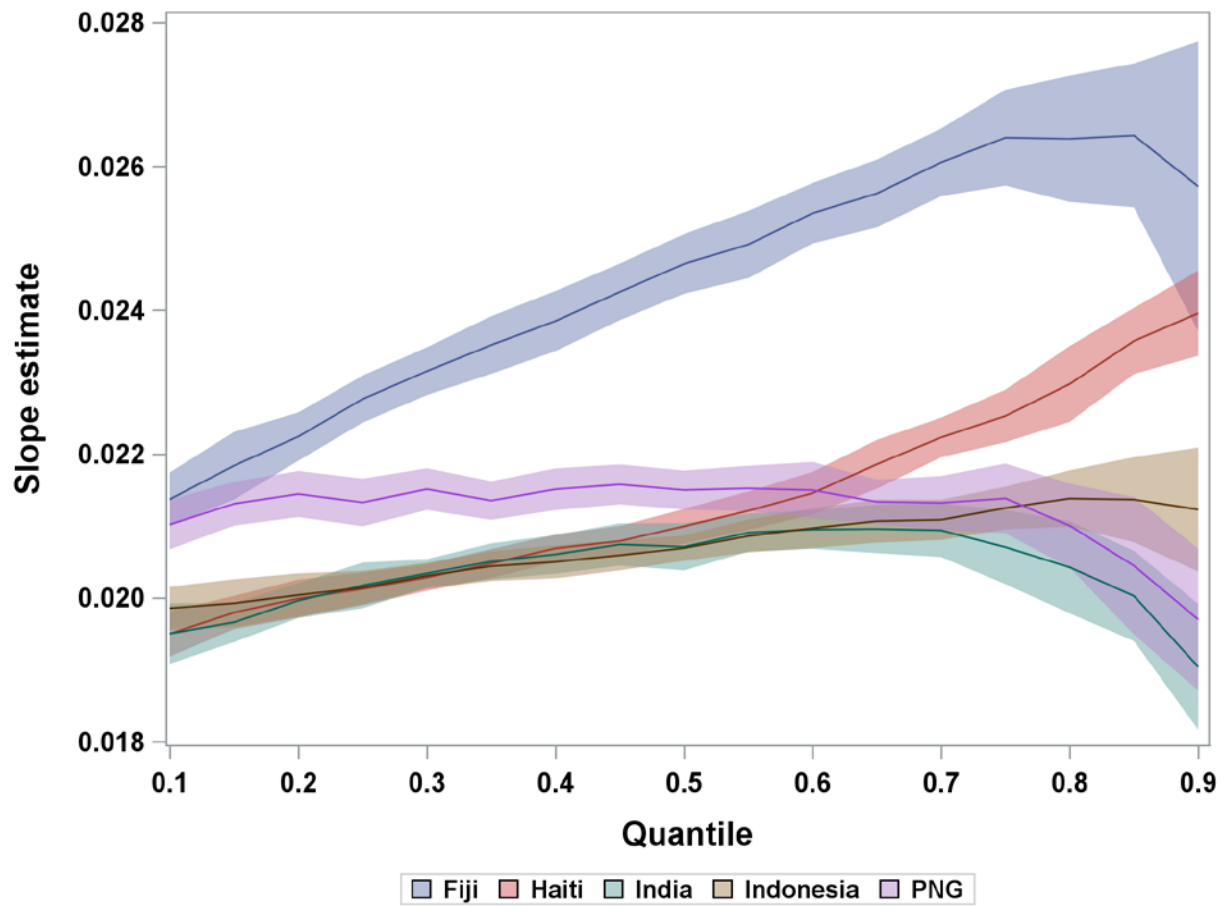

Supplement: S2 Fig — (PDF) [file pntd.0007541.s002.pdf]

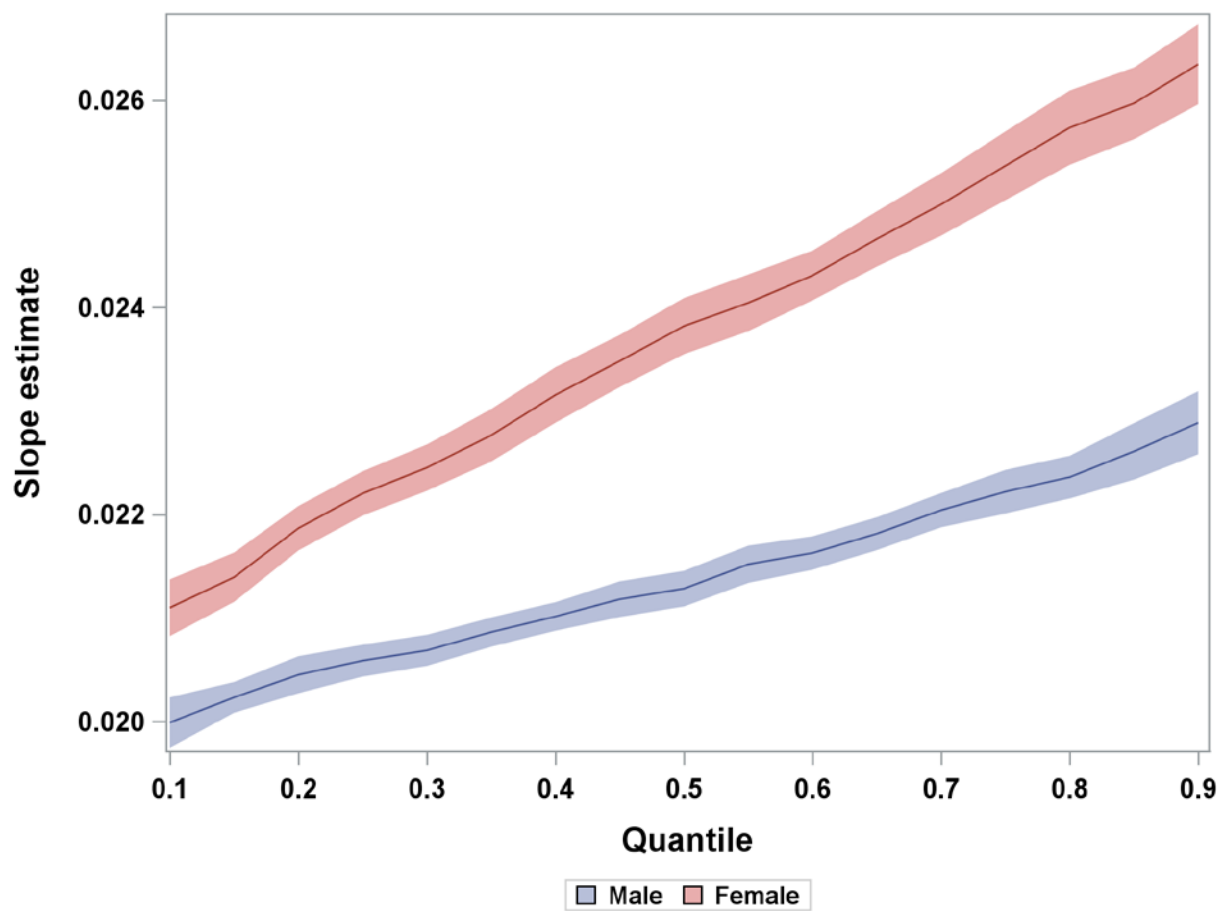

Supplement: S3 Fig — (PDF) [file pntd.0007541.s003.pdf]

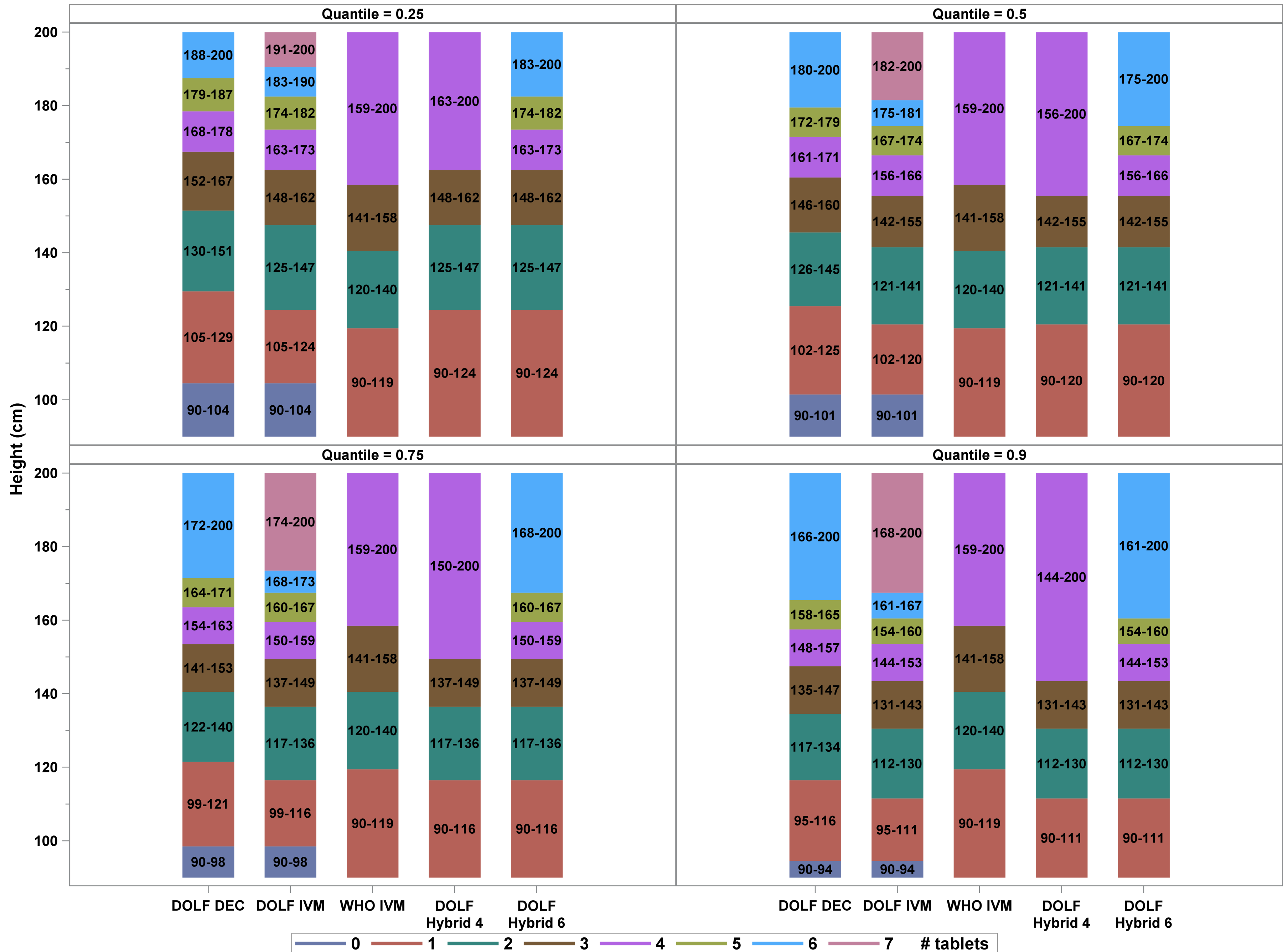

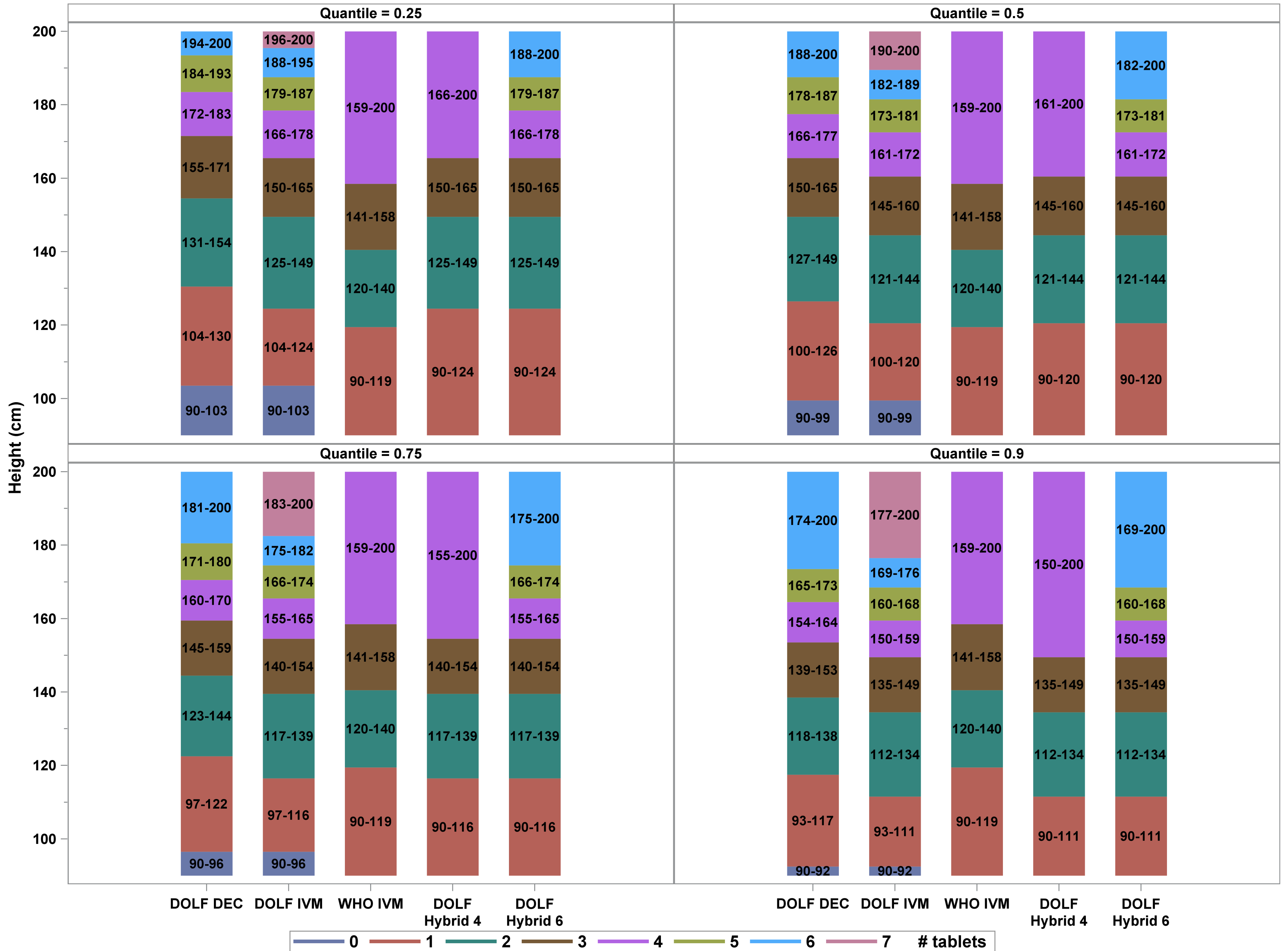

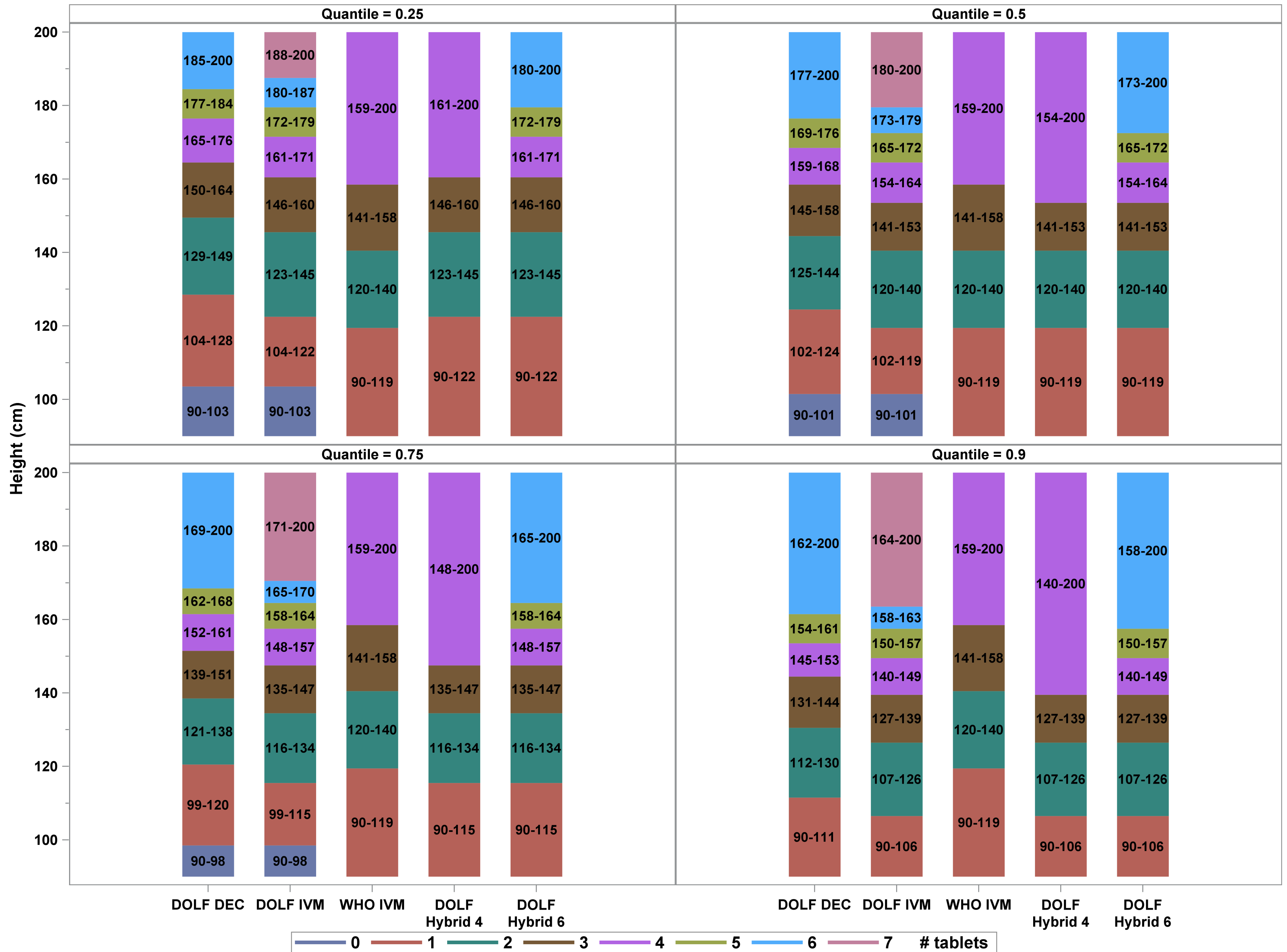

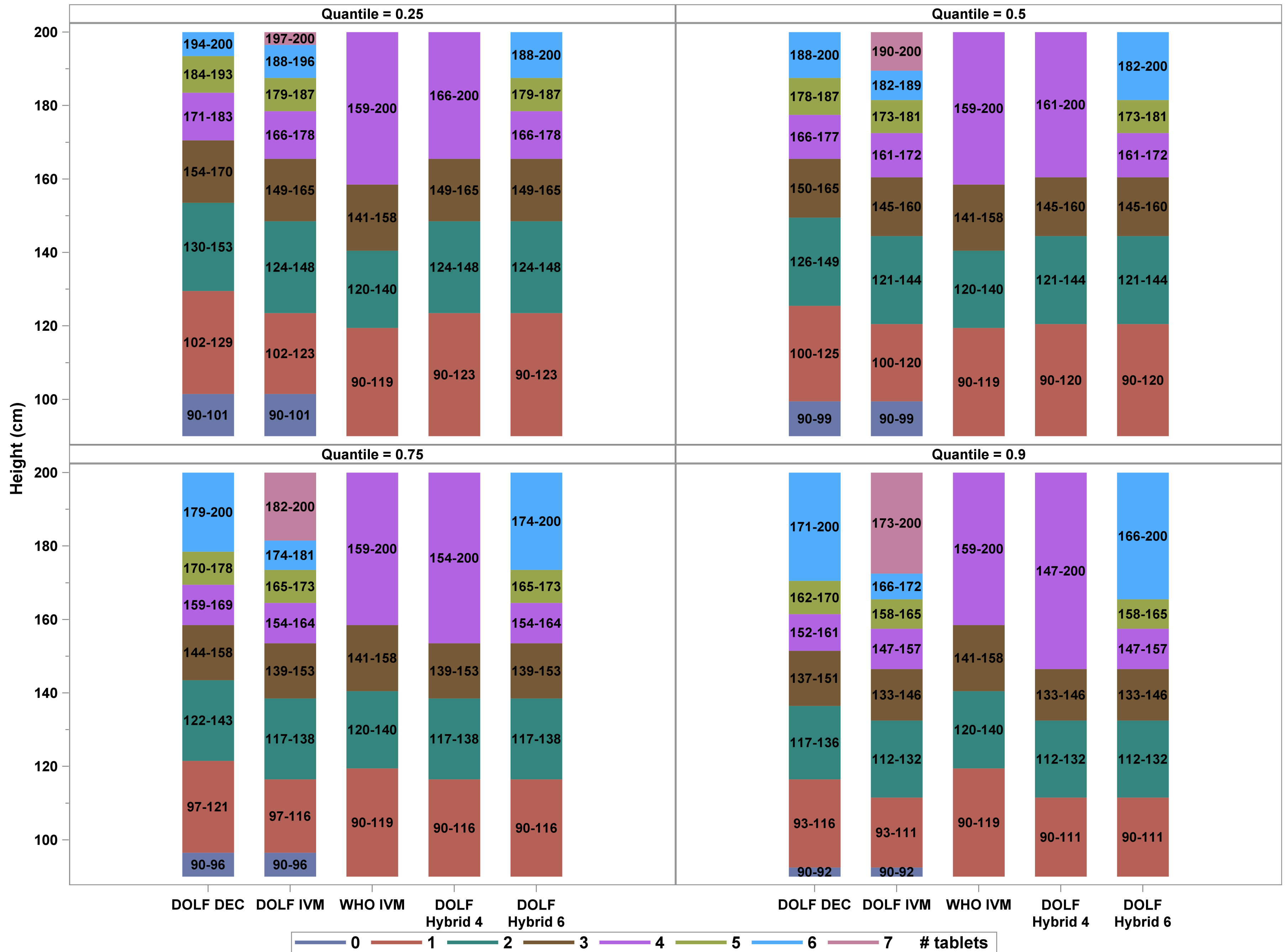

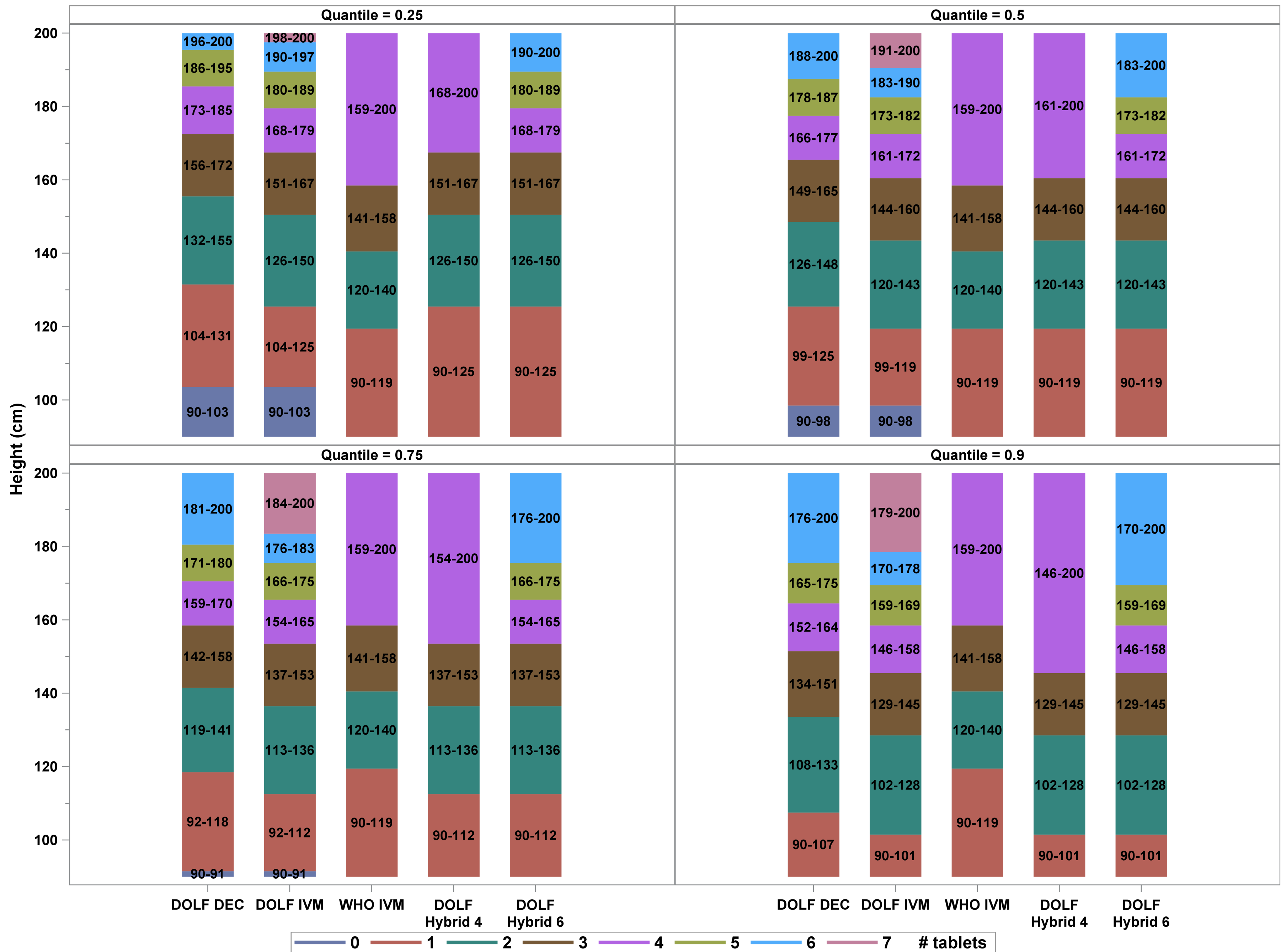

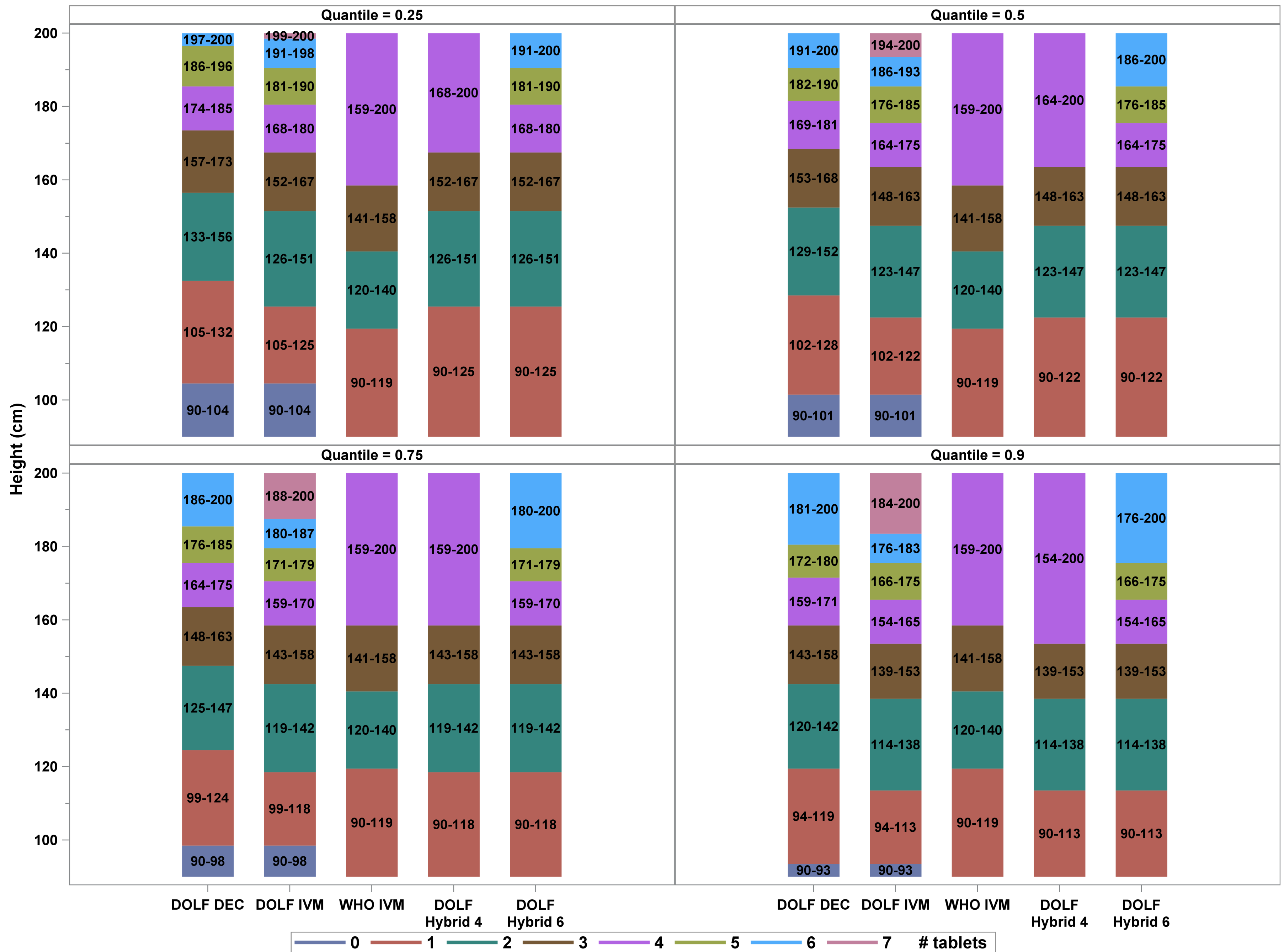

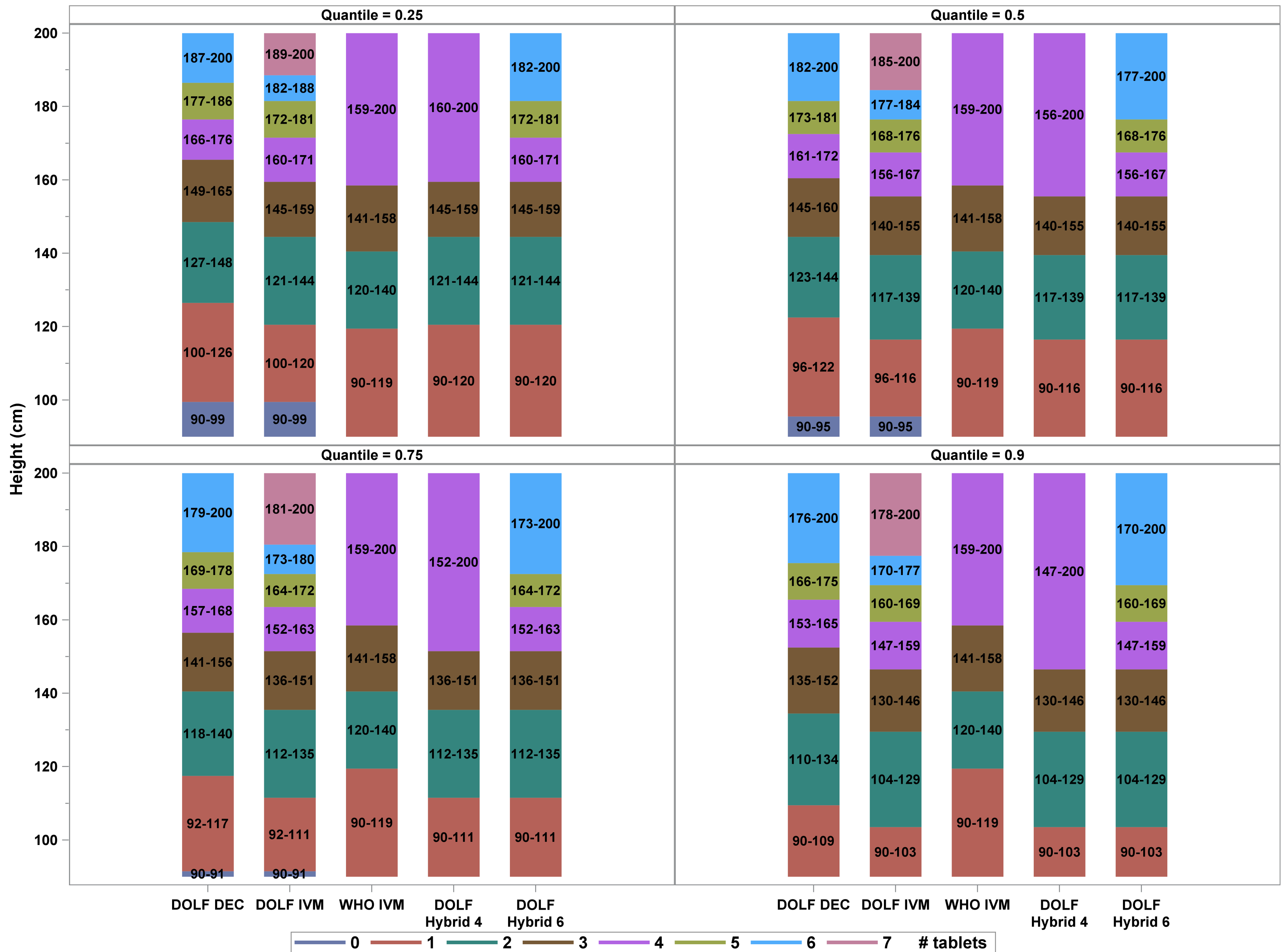

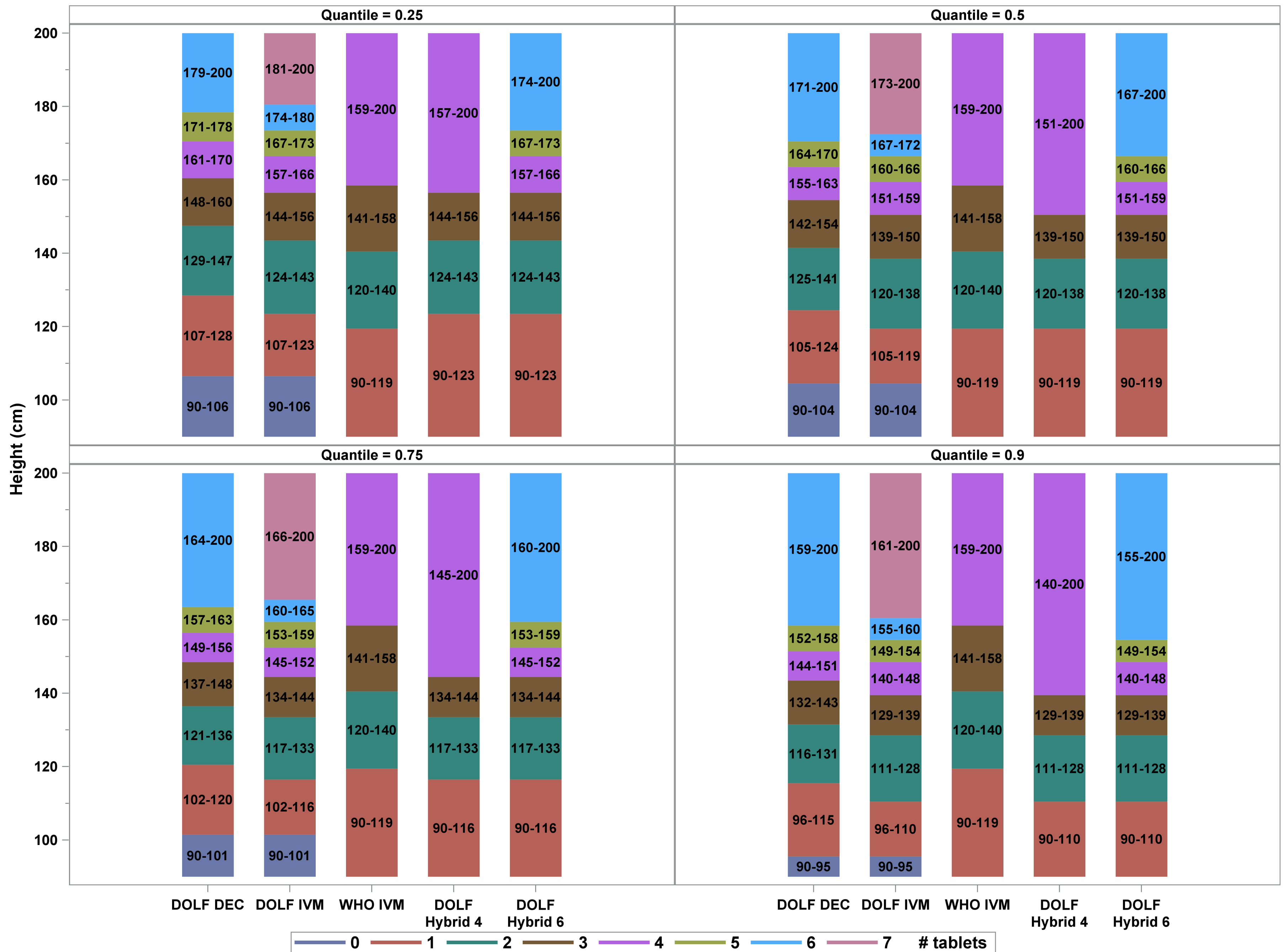

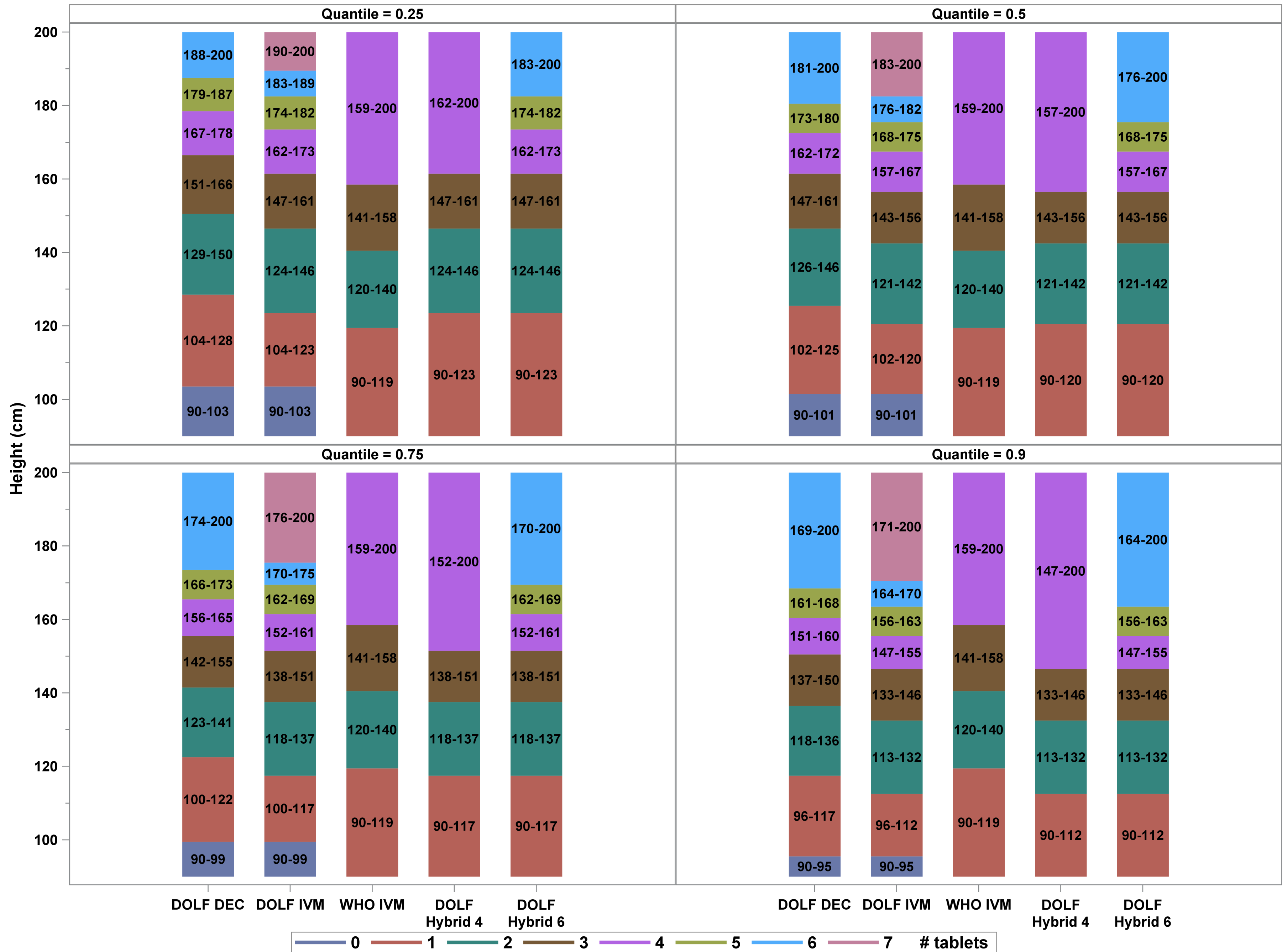

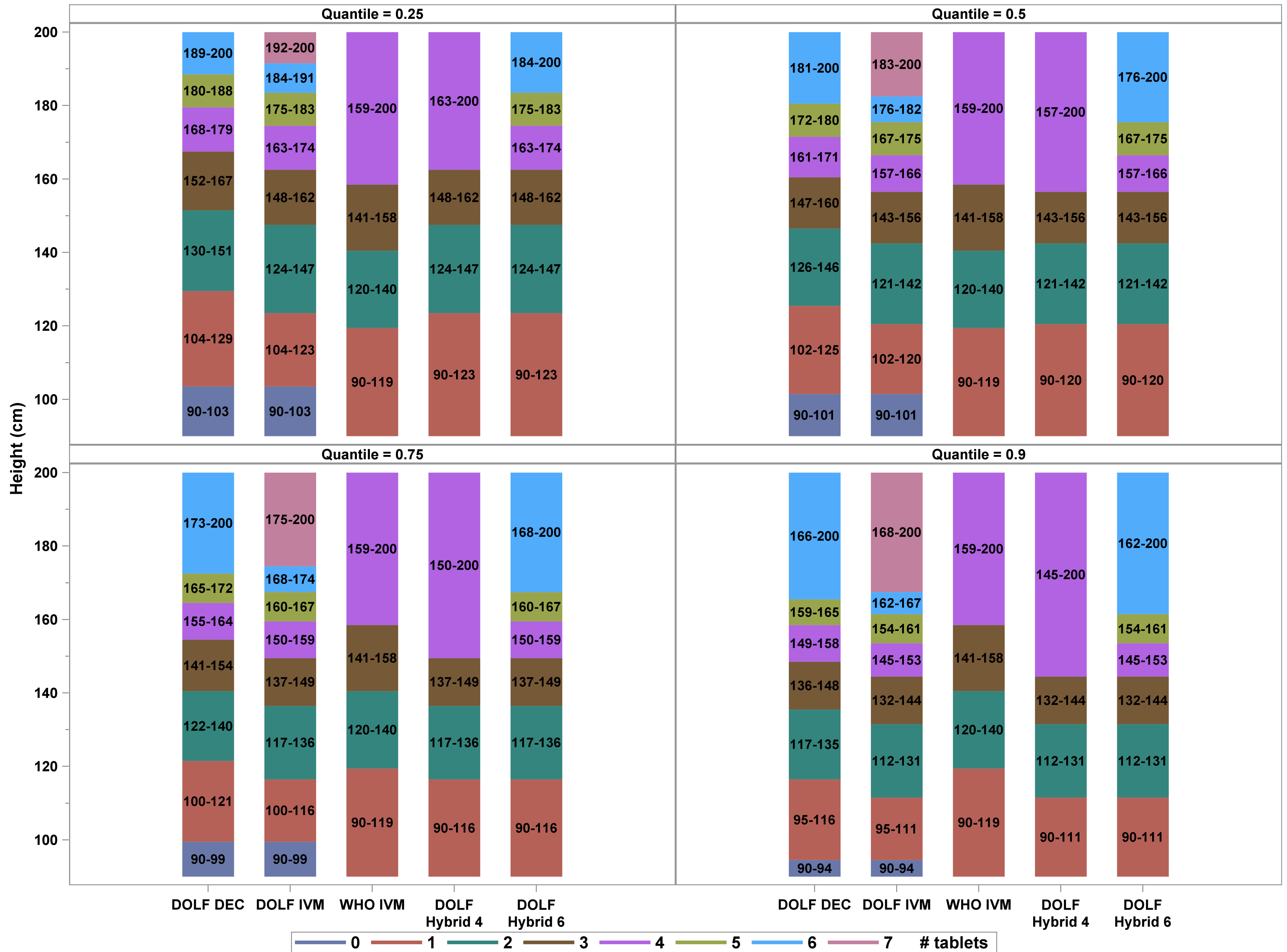

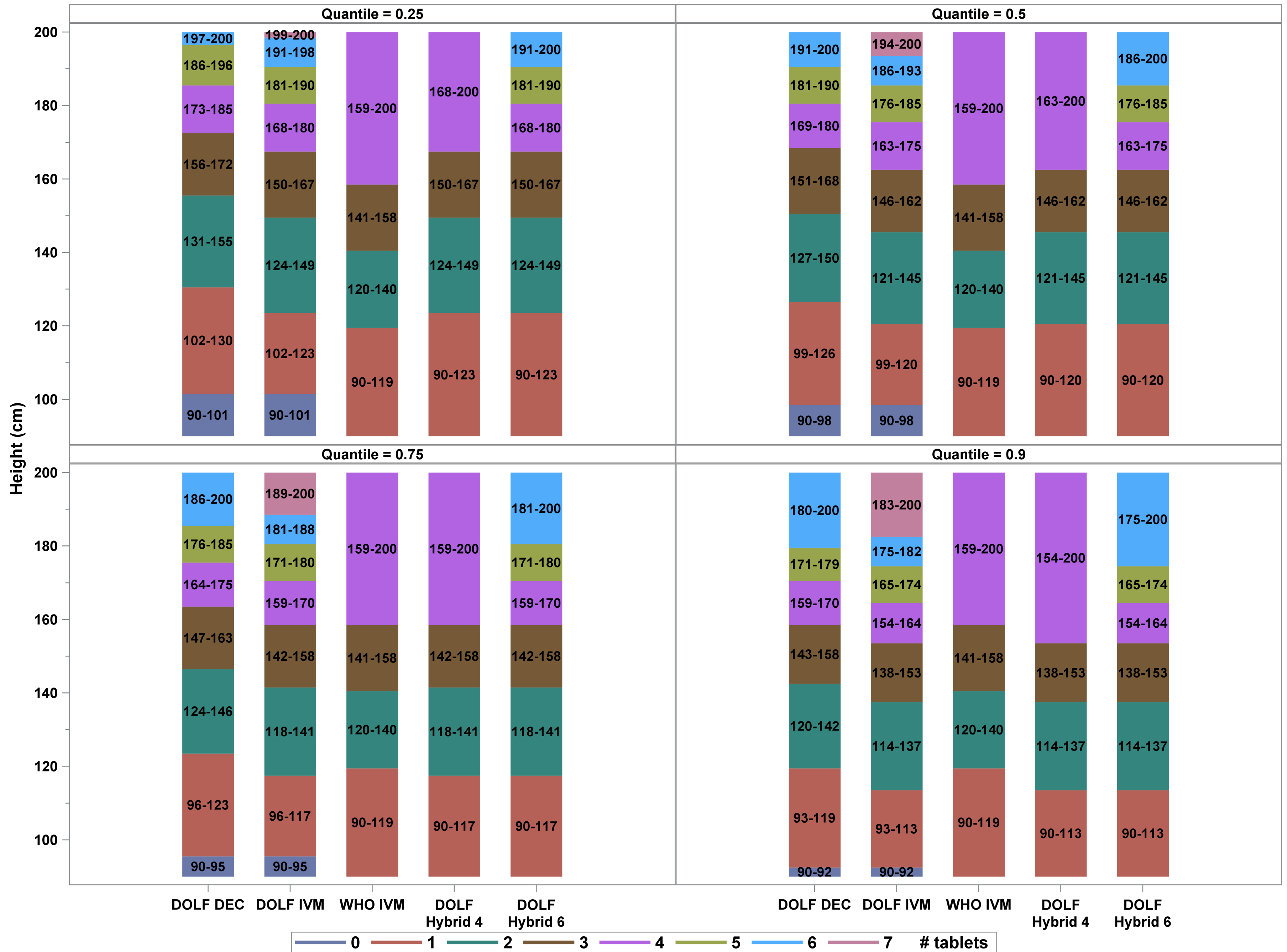

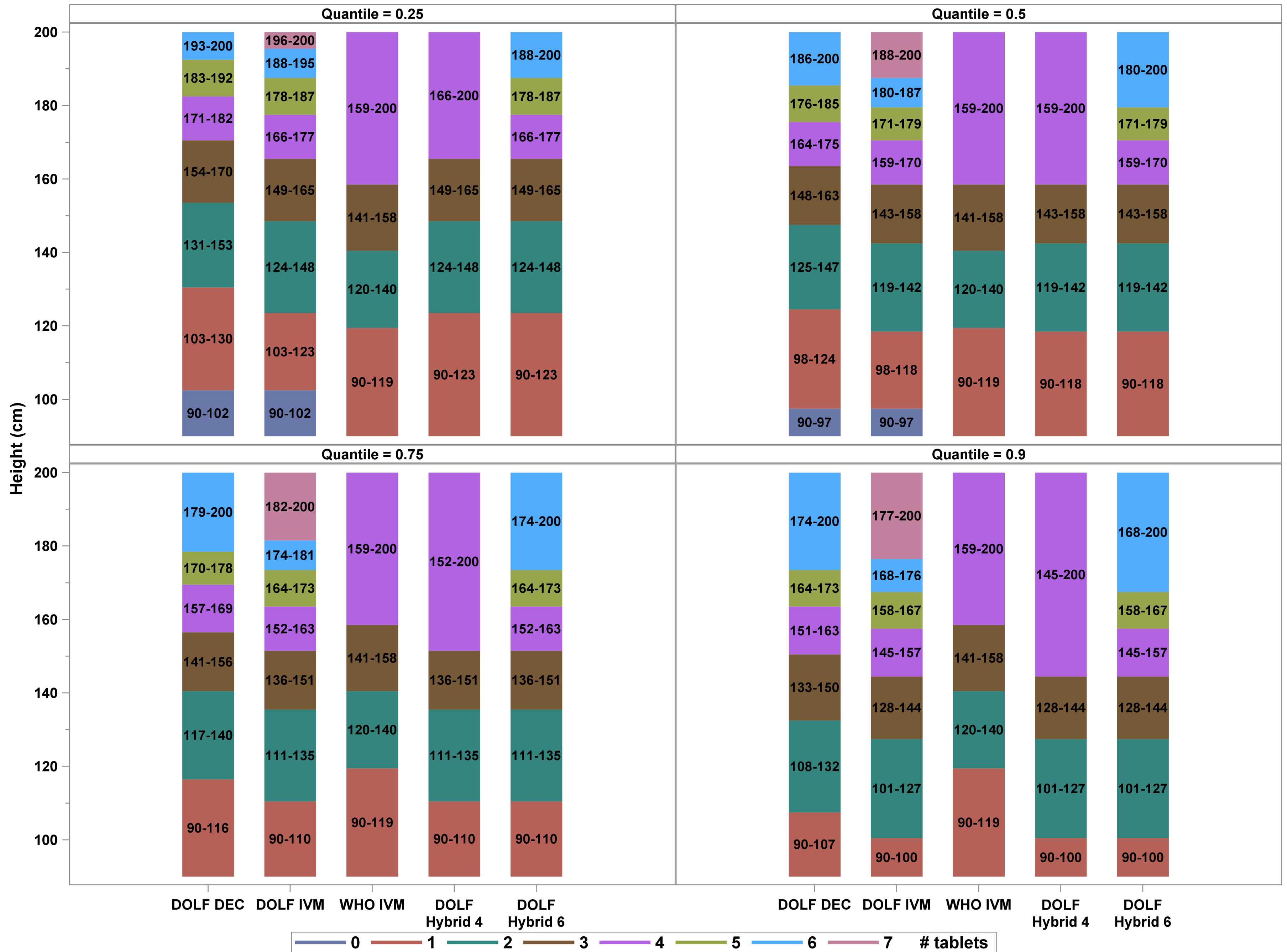

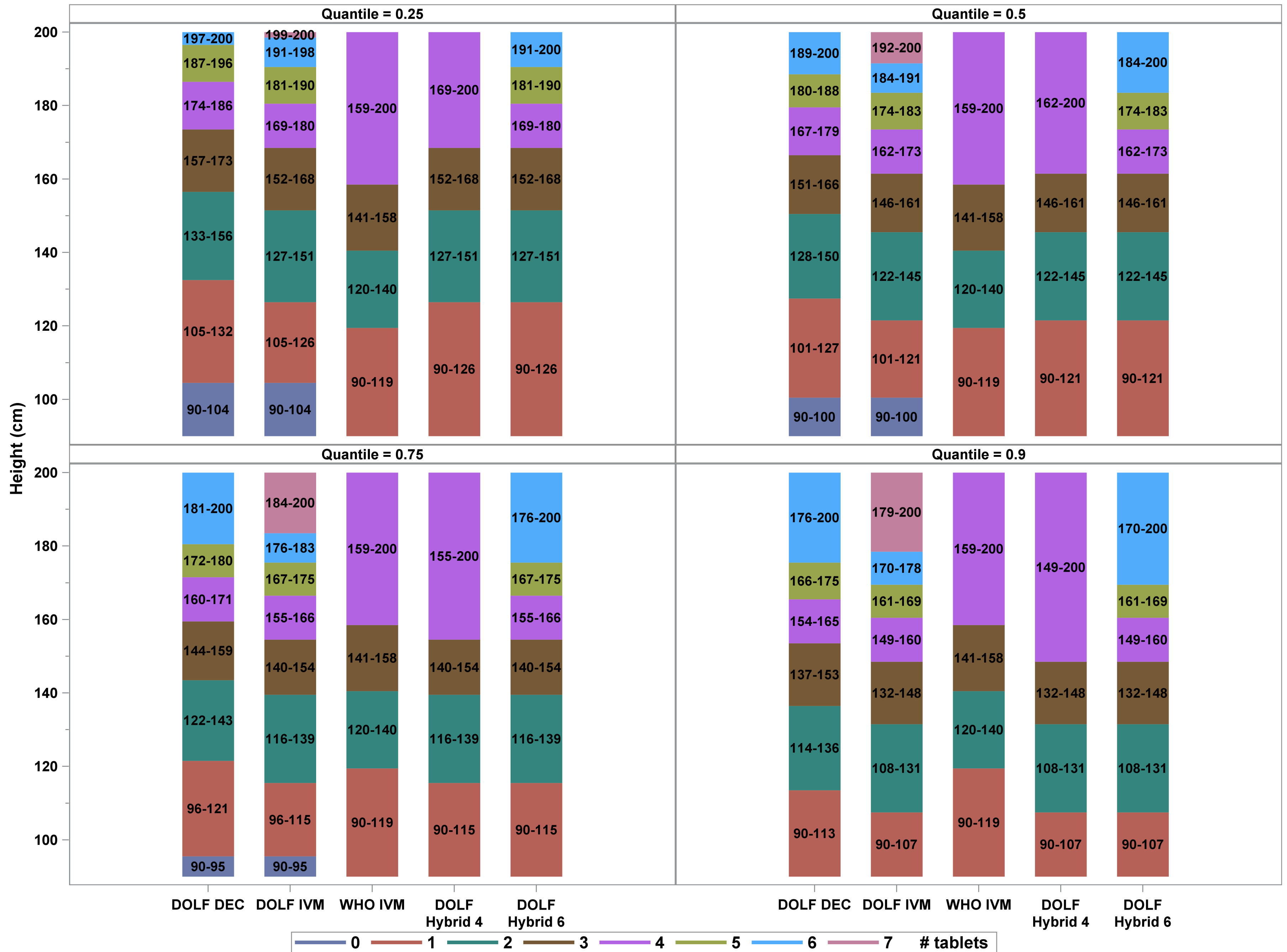

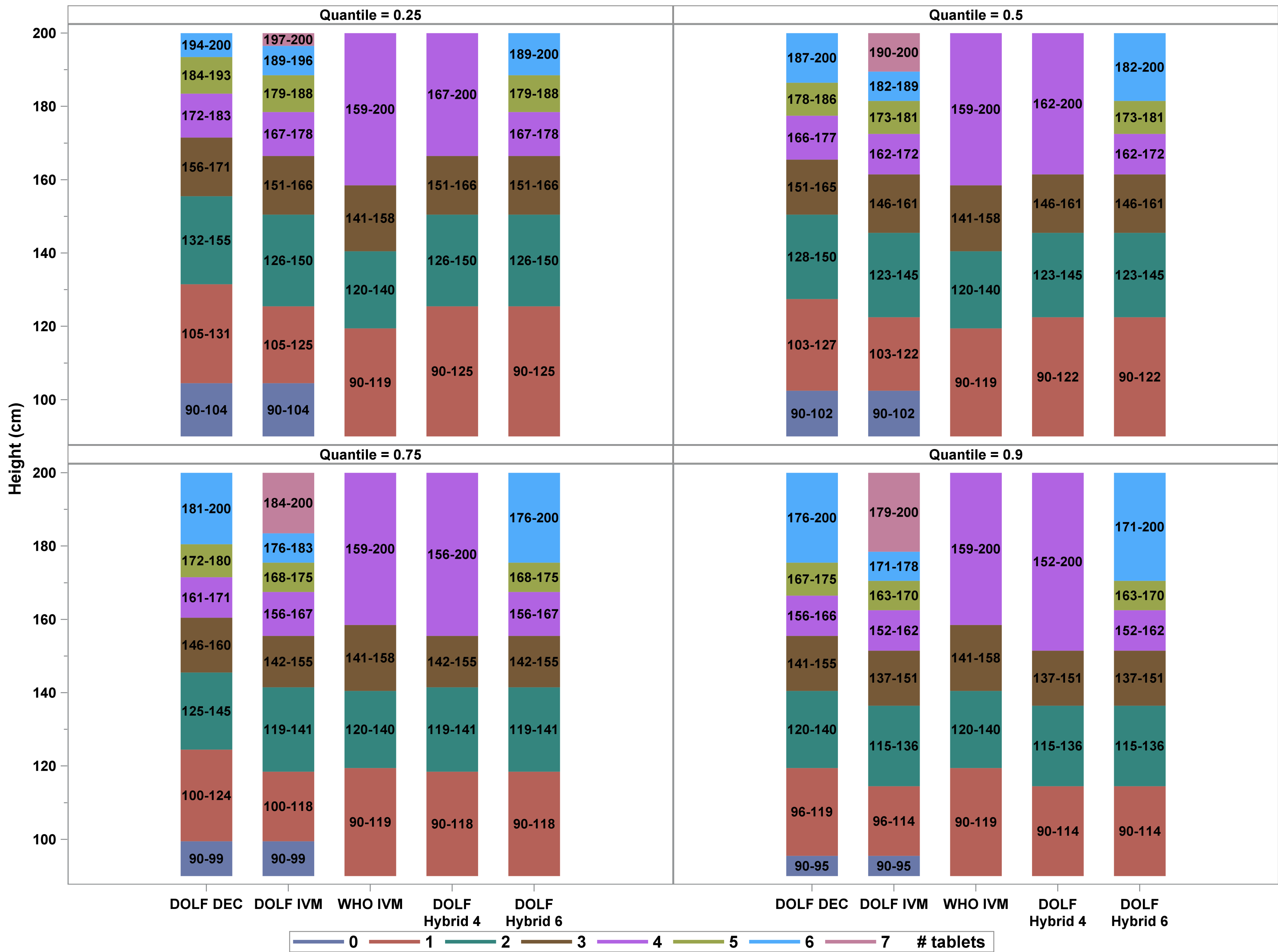

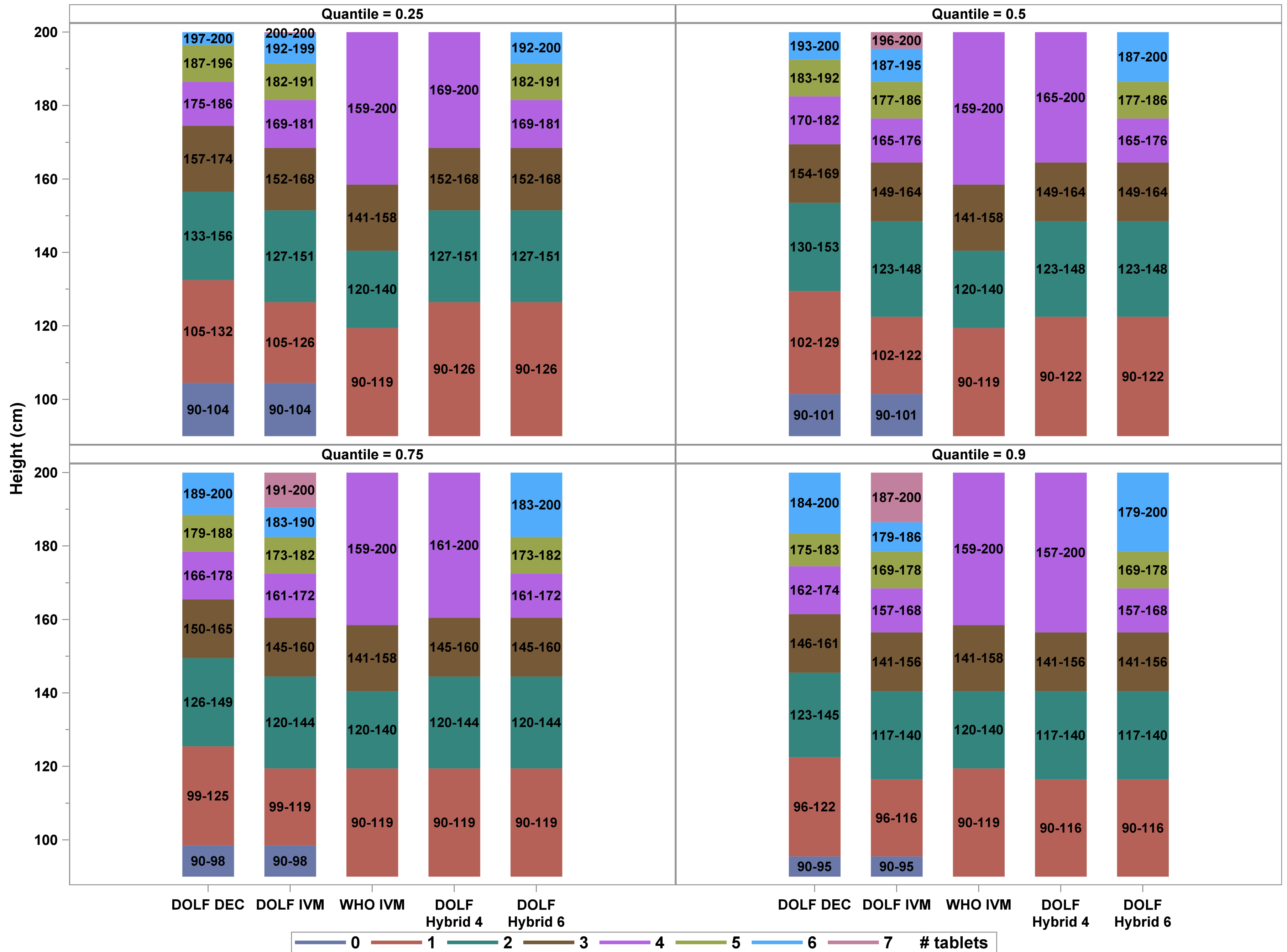

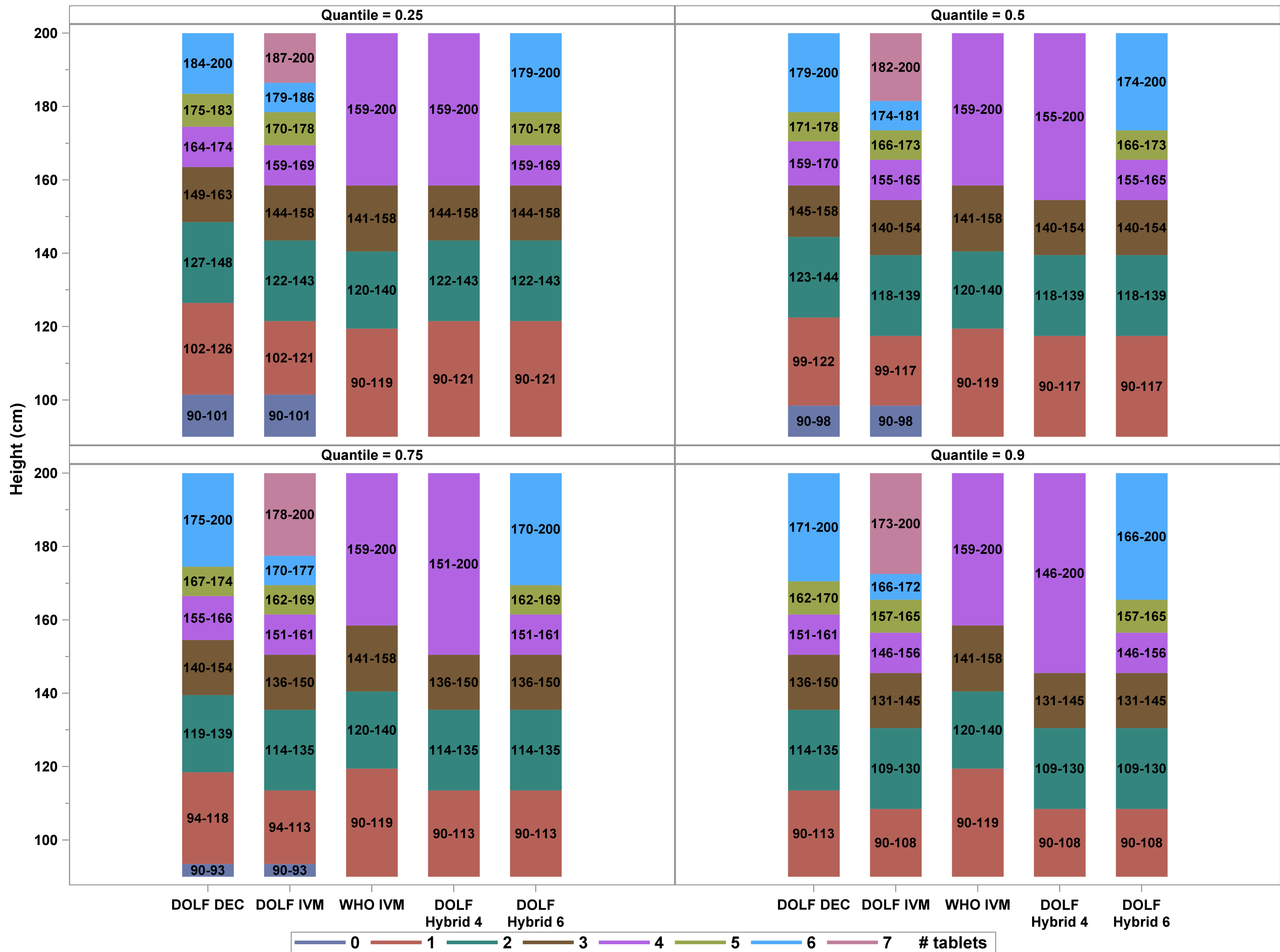

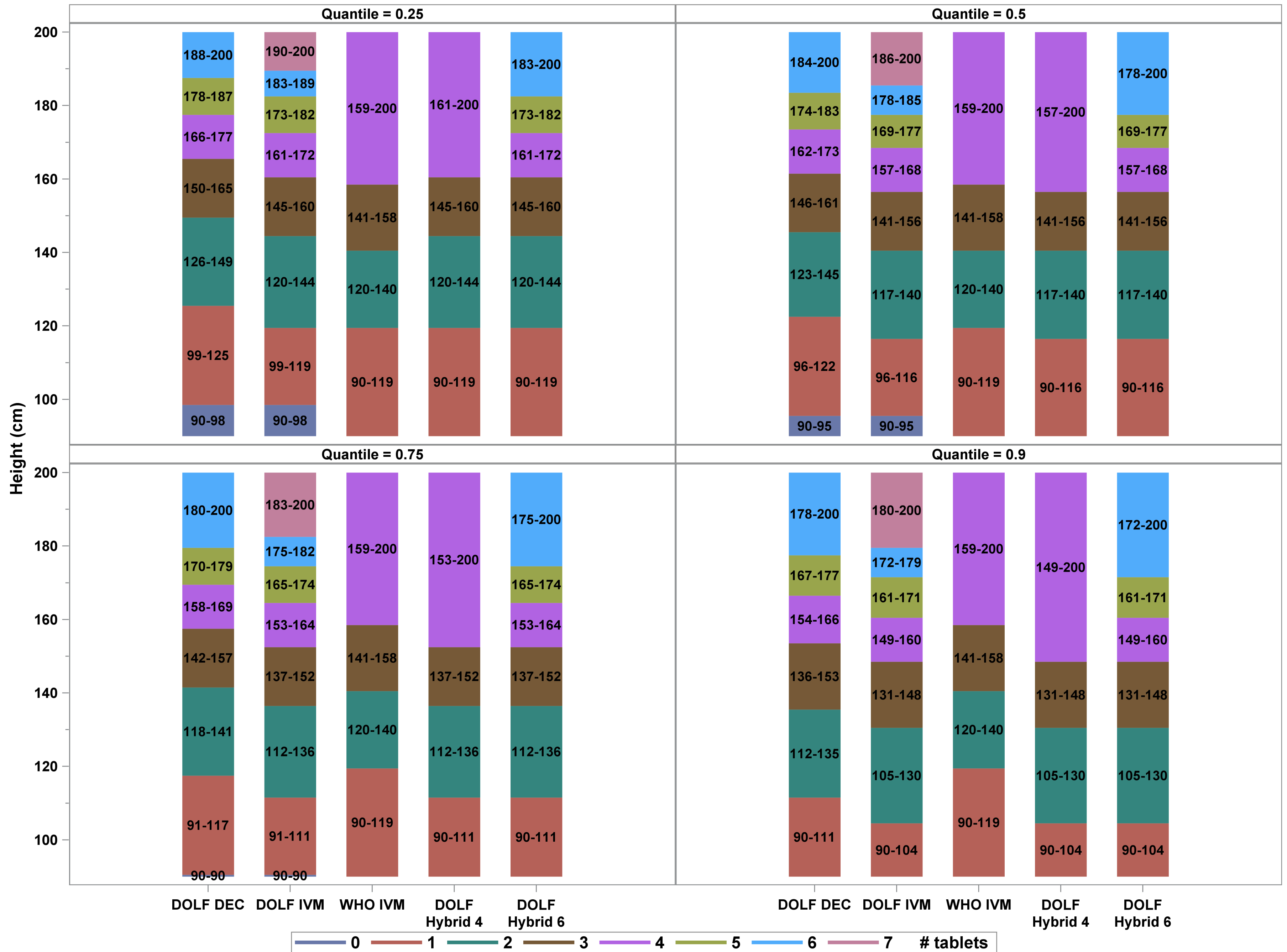

Supplement: S4 Fig — (PDF) [file pntd.0007541.s004.pdf]
