# Supplementary material for: Dosing pole recommendations for lymphatic filariasis elimination: A height-weight quantile regression modeling approach
Source: PLoS Negl Trop Dis. 2019 Jul 17;13(7):e0007541. doi: 10.1371/journal.pntd.0007541 (PMC6663033; doi:10.1371/journal.pntd.0007541)

IVM: Bar plot of percent of subjects below the recommended dose (BRD) for each strata and quantile

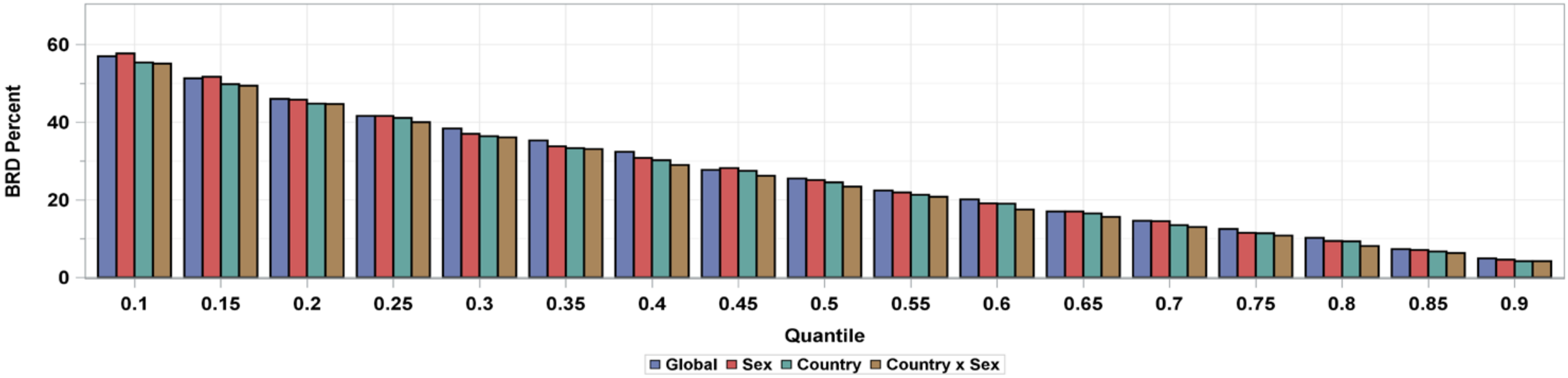


DEC: Bar plot of percent of subjects below the recommended dose (BRD) for each strata and quantile

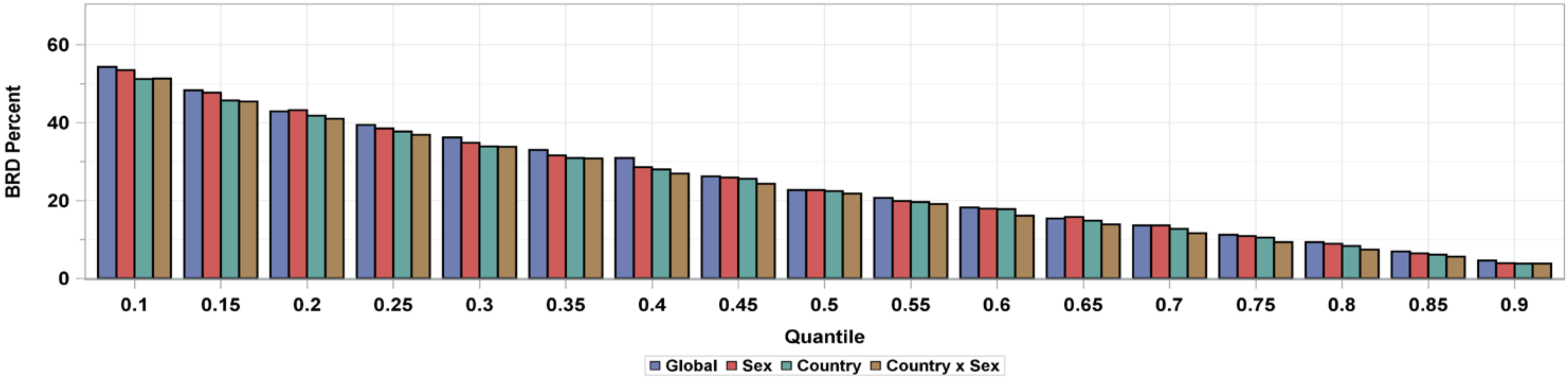

IVM: Bar plot of percent of subjects above the recommended dose (ARD) for each strata and quantile

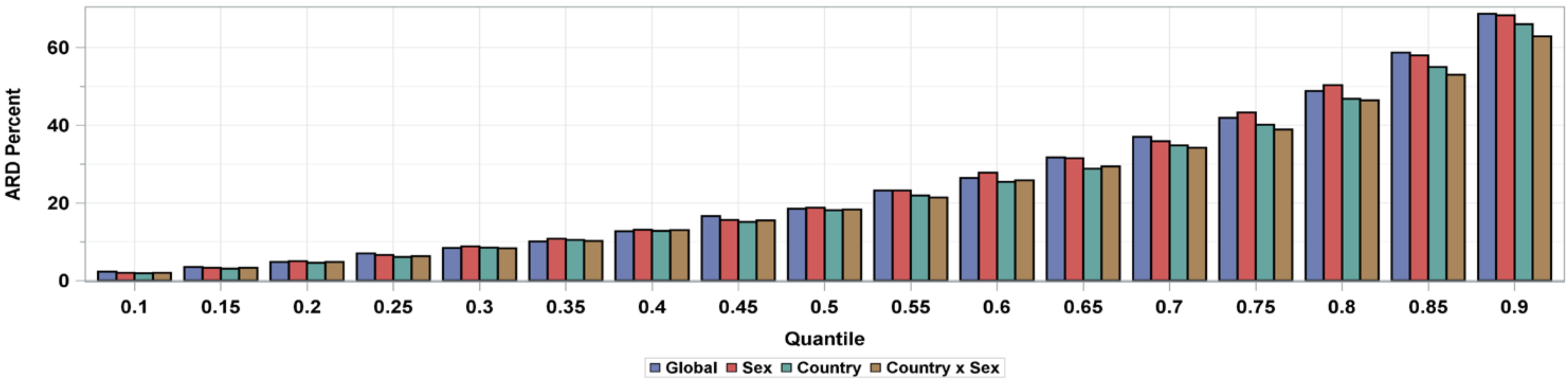


DEC: Bar plot of percent of subjects above the recommended dose (ARD) for each strata and quantile

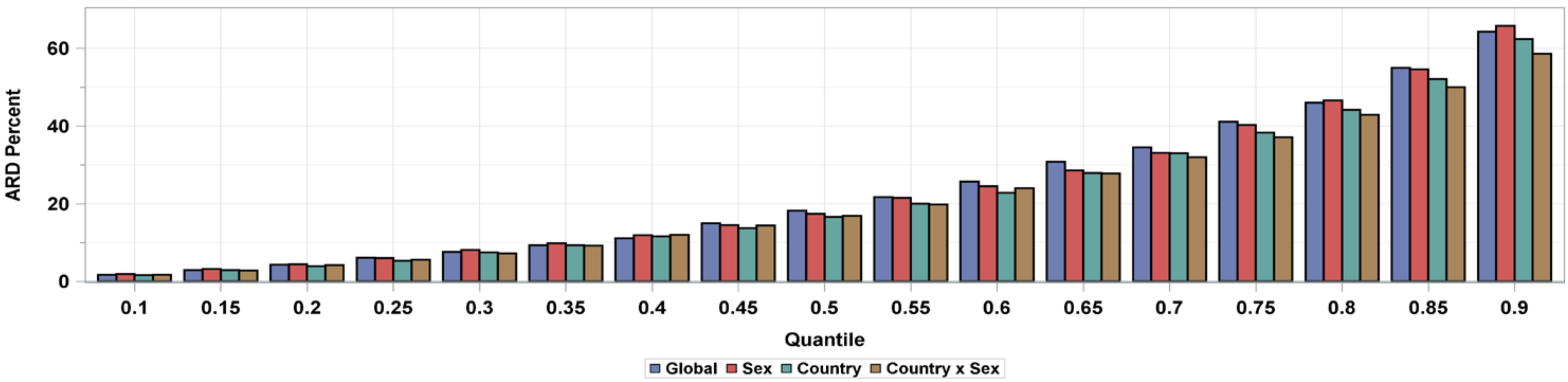

IVM: Bar plot of percent of subjects recommended the recommended dose (Recommended) for each strata and quantile

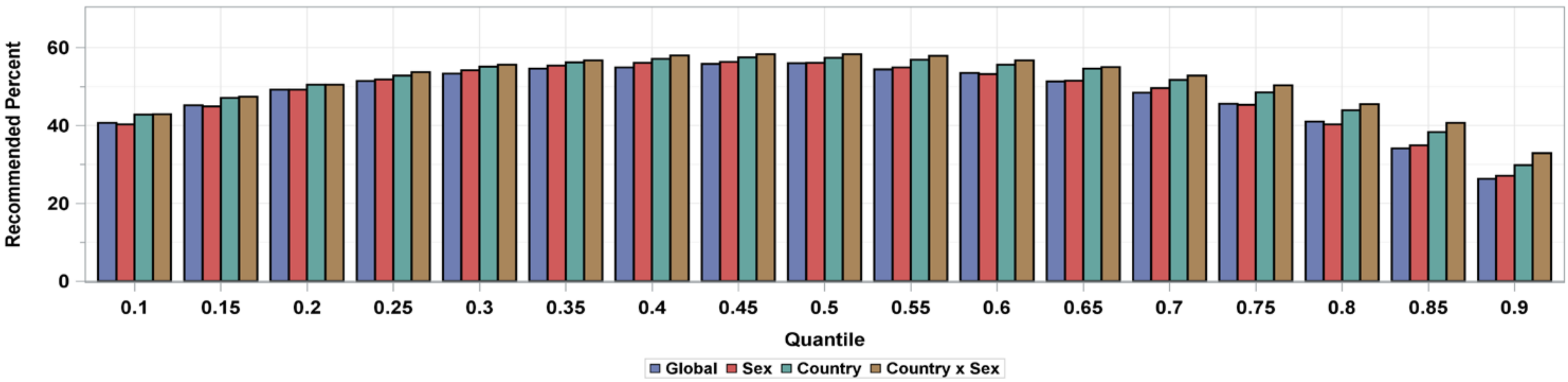


DEC: Bar plot of percent of subjects recommended the recommended dose (Recommended) for each strata and quantile

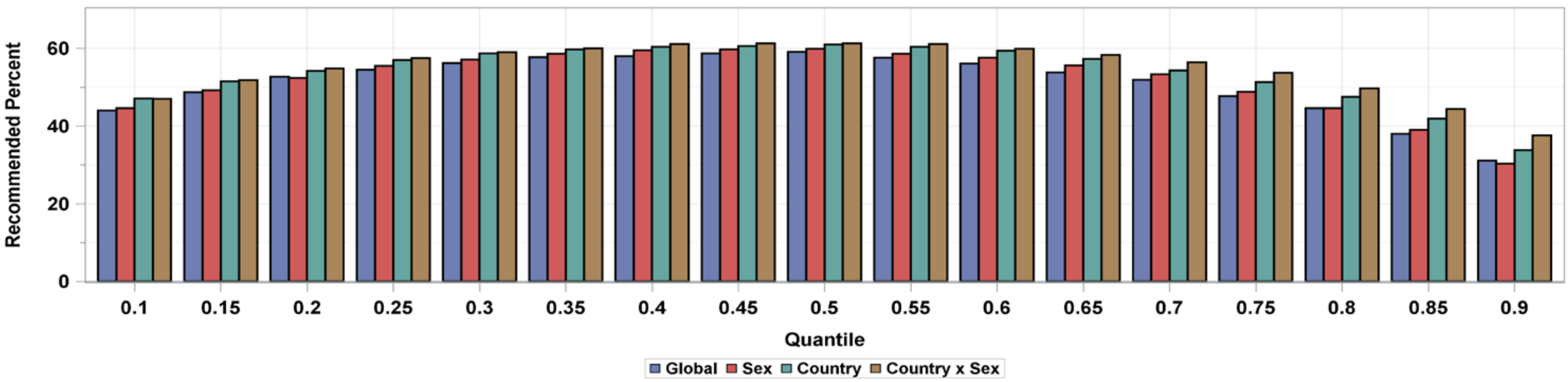

Supplement: S5 Fig — (PDF) [file pntd.0007541.s005.pdf]
