# Supplementary material for: Dosing pole recommendations for lymphatic filariasis elimination: A height-weight quantile regression modeling approach
Source: PLoS Negl Trop Dis. 2019 Jul 17;13(7):e0007541. doi: 10.1371/journal.pntd.0007541 (PMC6663033; doi:10.1371/journal.pntd.0007541)
Supplement: S1 Table — P-values correspond to a likelihood-ratio test that the slope coefficient = 0. All parameter estimates are presented on the log scale. Model predictions were obtained by taking the antilog of the model predictions. (PDF) [file pntd.0007541.s006.pdf]

Model group=1, Global

Scenario #	Quantile	Intercept	Height slope estimate	P-value
1	0.10	0.533 (0.505, 0.561)	0.02 (0.02, 0.02)	<.0001
2	0.15	0.518 (0.496, 0.54)	0.021 (0.02, 0.021)	<.0001
3	0.20	0.521 (0.501, 0.541)	0.021 (0.021, 0.021)	<.0001
4	0.25	0.517 (0.501, 0.533)	0.021 (0.021, 0.021)	<.0001
5	0.30	0.51 (0.496, 0.525)	0.021 (0.021, 0.021)	<.0001
6	0.35	0.505 (0.489, 0.52)	0.021 (0.021, 0.021)	<.0001
7	0.40	0.495 (0.476, 0.514)	0.022 (0.021, 0.022)	<.0001
8	0.45	0.493 (0.469, 0.516)	0.022 (0.022, 0.022)	<.0001
9	0.50	0.48 (0.46, 0.501)	0.022 (0.022, 0.022)	<.0001
10	0.55	0.481 (0.459, 0.503)	0.022 (0.022, 0.022)	<.0001
11	0.60	0.47 (0.449, 0.49)	0.022 (0.022, 0.023)	<.0001
12	0.65	0.462 (0.439, 0.485)	0.023 (0.022, 0.023)	<.0001
13	0.70	0.445 (0.418, 0.473)	0.023 (0.023, 0.023)	<.0001
14	0.75	0.444 (0.414, 0.474)	0.023 (0.023, 0.023)	<.0001
15	0.80	0.439 (0.402, 0.475)	0.023 (0.023, 0.024)	<.0001
16	0.85	0.425 (0.39, 0.459)	0.024 (0.024, 0.024)	<.0001
17	0.90	0.43 (0.382, 0.478)	0.024 (0.024, 0.025)	<.0001

Model group=2, Country: Fiji

Scenario #	Quantile	Intercept	Height slope estimate	P-value
18	0.10	0.437 (0.375, 0.499)	0.021 (0.021, 0.022)	<.0001
19	0.15	0.398 (0.327, 0.47)	0.022 (0.021, 0.022)	<.0001
20	0.20	0.369 (0.312, 0.425)	0.022 (0.022, 0.023)	<.0001
21	0.25	0.317 (0.265, 0.368)	0.023 (0.022, 0.023)	<.0001
22	0.30	0.285 (0.229, 0.342)	0.023 (0.023, 0.024)	<.0001
23	0.35	0.257 (0.188, 0.326)	0.024 (0.023, 0.024)	<.0001
24	0.40	0.234 (0.171, 0.296)	0.024 (0.023, 0.024)	<.0001
25	0.45	0.199 (0.132, 0.266)	0.024 (0.024, 0.025)	<.0001
26	0.50	0.166 (0.104, 0.229)	0.025 (0.024, 0.025)	<.0001
27	0.55	0.154 (0.09, 0.218)	0.025 (0.024, 0.025)	<.0001
28	0.60	0.116 (0.055, 0.178)	0.025 (0.025, 0.026)	<.0001
29	0.65	0.109 (0.041, 0.177)	0.026 (0.025, 0.026)	<.0001
30	0.70	0.077 (0.004, 0.151)	0.026 (0.026, 0.027)	<.0001
31	0.75	0.066 (-0.034, 0.167)	0.026 (0.026, 0.027)	<.0001
32	0.80	0.116 (-0.022, 0.254)	0.026 (0.026, 0.027)	<.0001
33	0.85	0.164 (-0.005, 0.332)	0.026 (0.025, 0.027)	<.0001
34	0.90	0.364 (0.083, 0.646)	0.026 (0.024, 0.027)	<.0001

Model group=2, Country: Haiti

Scenario #	Quantile	Intercept	Height slope estimate	P-value
35	0.10	0.643 (0.6, 0.687)	0.02 (0.019, 0.02)	<.0001
36	0.15	0.631 (0.596, 0.666)	0.02 (0.02, 0.02)	<.0001
37	0.20	0.628 (0.593, 0.662)	0.02 (0.02, 0.02)	<.0001
38	0.25	0.629 (0.595, 0.664)	0.02 (0.02, 0.02)	<.0001
39	0.30	0.626 (0.595, 0.656)	0.02 (0.02, 0.02)	<.0001
40	0.35	0.615 (0.582, 0.647)	0.02 (0.02, 0.021)	<.0001
41	0.40	0.602 (0.568, 0.635)	0.021 (0.02, 0.021)	<.0001
42	0.45	0.605 (0.572, 0.638)	0.021 (0.021, 0.021)	<.0001
43	0.50	0.593 (0.559, 0.628)	0.021 (0.021, 0.021)	<.0001
44	0.55	0.584 (0.539, 0.628)	0.021 (0.021, 0.022)	<.0001

45	0.60	0.569 (0.527, 0.611)	0.021 (0.021, 0.022)	<.0001
46	0.65	0.537 (0.491, 0.583)	0.022 (0.022, 0.022)	<.0001
47	0.70	0.511 (0.473, 0.549)	0.022 (0.022, 0.022)	<.0001
48	0.75	0.499 (0.445, 0.554)	0.023 (0.022, 0.023)	<.0001
49	0.80	0.474 (0.401, 0.547)	0.023 (0.022, 0.023)	<.0001
50	0.85	0.439 (0.373, 0.504)	0.024 (0.023, 0.024)	<.0001
51	0.90	0.448 (0.363, 0.532)	0.024 (0.023, 0.025)	<.0001

Model group=2, Country: India

Scenario #	Quantile	Intercept	Height slope estimate	P-value
52	0.10	0.582 (0.525, 0.64)	0.02 (0.019, 0.02)	<.0001
53	0.15	0.604 (0.566, 0.643)	0.02 (0.019, 0.02)	<.0001
54	0.20	0.595 (0.553, 0.637)	0.02 (0.02, 0.02)	<.0001
55	0.25	0.591 (0.544, 0.637)	0.02 (0.02, 0.02)	<.0001
56	0.30	0.595 (0.567, 0.623)	0.02 (0.02, 0.021)	<.0001
57	0.35	0.595 (0.56, 0.63)	0.021 (0.02, 0.021)	<.0001
58	0.40	0.608 (0.565, 0.65)	0.021 (0.02, 0.021)	<.0001
59	0.45	0.61 (0.567, 0.654)	0.021 (0.02, 0.021)	<.0001
60	0.50	0.643 (0.597, 0.688)	0.021 (0.02, 0.021)	<.0001
61	0.55	0.643 (0.604, 0.683)	0.021 (0.021, 0.021)	<.0001
62	0.60	0.663 (0.624, 0.701)	0.021 (0.021, 0.021)	<.0001
63	0.65	0.689 (0.635, 0.742)	0.021 (0.021, 0.021)	<.0001
64	0.70	0.725 (0.668, 0.782)	0.021 (0.021, 0.021)	<.0001
65	0.75	0.788 (0.709, 0.867)	0.021 (0.02, 0.021)	<.0001
66	0.80	0.869 (0.773, 0.965)	0.02 (0.02, 0.021)	<.0001
67	0.85	0.977 (0.885, 1.07)	0.02 (0.019, 0.021)	<.0001
68	0.90	1.187 (1.06, 1.314)	0.019 (0.018, 0.02)	<.0001

Model group=2, Country: Indonesia

Scenario #	Quantile	Intercept	Height slope estimate	P-value
69	0.10	0.55 (0.506, 0.594)	0.02 (0.02, 0.02)	<.0001
70	0.15	0.567 (0.516, 0.617)	0.02 (0.02, 0.02)	<.0001
71	0.20	0.573 (0.529, 0.617)	0.02 (0.02, 0.02)	<.0001
72	0.25	0.578 (0.547, 0.61)	0.02 (0.02, 0.02)	<.0001
73	0.30	0.573 (0.546, 0.599)	0.02 (0.02, 0.02)	<.0001
74	0.35	0.568 (0.536, 0.6)	0.02 (0.02, 0.021)	<.0001
75	0.40	0.575 (0.54, 0.611)	0.021 (0.02, 0.021)	<.0001
76	0.45	0.579 (0.545, 0.613)	0.021 (0.02, 0.021)	<.0001
77	0.50	0.577 (0.551, 0.603)	0.021 (0.021, 0.021)	<.0001
78	0.55	0.568 (0.534, 0.601)	0.021 (0.021, 0.021)	<.0001
79	0.60	0.569 (0.528, 0.61)	0.021 (0.021, 0.021)	<.0001
80	0.65	0.574 (0.531, 0.618)	0.021 (0.021, 0.021)	<.0001
81	0.70	0.591 (0.549, 0.633)	0.021 (0.021, 0.021)	<.0001
82	0.75	0.59 (0.549, 0.631)	0.021 (0.021, 0.022)	<.0001
83	0.80	0.598 (0.54, 0.655)	0.021 (0.021, 0.022)	<.0001
84	0.85	0.63 (0.552, 0.709)	0.021 (0.021, 0.022)	<.0001
85	0.90	0.694 (0.576, 0.811)	0.021 (0.02, 0.022)	<.0001

Model group=2, Country: PNG

Scenario #	Quantile	Intercept	Height slope estimate	P-value
86	0.10	0.512 (0.456, 0.568)	0.021 (0.021, 0.021)	<.0001
87	0.15	0.503 (0.456, 0.549)	0.021 (0.021, 0.022)	<.0001

88	0.20	0.508 (0.457, 0.56)	0.021 (0.021, 0.022)	<.0001
89	0.25	0.549 (0.497, 0.602)	0.021 (0.021, 0.022)	<.0001
90	0.30	0.54 (0.489, 0.592)	0.022 (0.021, 0.022)	<.0001
91	0.35	0.583 (0.534, 0.631)	0.021 (0.021, 0.022)	<.0001
92	0.40	0.578 (0.534, 0.622)	0.022 (0.021, 0.022)	<.0001
93	0.45	0.586 (0.542, 0.63)	0.022 (0.021, 0.022)	<.0001
94	0.50	0.615 (0.567, 0.664)	0.021 (0.021, 0.022)	<.0001
95	0.55	0.628 (0.578, 0.679)	0.022 (0.021, 0.022)	<.0001
96	0.60	0.649 (0.591, 0.706)	0.021 (0.021, 0.022)	<.0001
97	0.65	0.692 (0.646, 0.737)	0.021 (0.021, 0.022)	<.0001
98	0.70	0.71 (0.652, 0.769)	0.021 (0.021, 0.022)	<.0001
99	0.75	0.719 (0.645, 0.793)	0.021 (0.021, 0.022)	<.0001
100	0.80	0.801 (0.711, 0.89)	0.021 (0.02, 0.022)	<.0001
101	0.85	0.918 (0.766, 1.07)	0.02 (0.019, 0.021)	<.0001
102	0.90	1.074 (0.914, 1.233)	0.02 (0.019, 0.021)	<.0001

Model group=3, Sex: Female

Scenario #	Quantile	Intercept	Height slope estimate	P-value
103	0.10	0.415 (0.374, 0.455)	0.021 (0.021, 0.021)	<.0001
104	0.15	0.415 (0.379, 0.452)	0.021 (0.021, 0.022)	<.0001
105	0.20	0.377 (0.346, 0.407)	0.022 (0.022, 0.022)	<.0001
106	0.25	0.356 (0.326, 0.385)	0.022 (0.022, 0.022)	<.0001
107	0.30	0.346 (0.314, 0.378)	0.022 (0.022, 0.023)	<.0001
108	0.35	0.324 (0.29, 0.358)	0.023 (0.023, 0.023)	<.0001
109	0.40	0.293 (0.254, 0.332)	0.023 (0.023, 0.023)	<.0001
110	0.45	0.272 (0.238, 0.305)	0.023 (0.023, 0.024)	<.0001
111	0.50	0.249 (0.211, 0.286)	0.024 (0.024, 0.024)	<.0001
112	0.55	0.241 (0.203, 0.28)	0.024 (0.024, 0.024)	<.0001
113	0.60	0.228 (0.193, 0.263)	0.024 (0.024, 0.025)	<.0001
114	0.65	0.204 (0.164, 0.244)	0.025 (0.024, 0.025)	<.0001
115	0.70	0.186 (0.148, 0.224)	0.025 (0.025, 0.025)	<.0001
116	0.75	0.167 (0.124, 0.211)	0.025 (0.025, 0.026)	<.0001
117	0.80	0.154 (0.103, 0.205)	0.026 (0.025, 0.026)	<.0001
118	0.85	0.169 (0.12, 0.219)	0.026 (0.026, 0.026)	<.0001
119	0.90	0.178 (0.126, 0.23)	0.026 (0.026, 0.027)	<.0001

Model group=3, Sex: Male

Scenario #	Quantile	Intercept	Height slope estimate	P-value
120	0.10	0.546 (0.51, 0.581)	0.02 (0.02, 0.02)	<.0001
121	0.15	0.547 (0.526, 0.568)	0.02 (0.02, 0.02)	<.0001
122	0.20	0.541 (0.515, 0.568)	0.02 (0.02, 0.021)	<.0001
123	0.25	0.547 (0.522, 0.572)	0.021 (0.02, 0.021)	<.0001
124	0.30	0.555 (0.531, 0.578)	0.021 (0.021, 0.021)	<.0001
125	0.35	0.547 (0.526, 0.568)	0.021 (0.021, 0.021)	<.0001
126	0.40	0.545 (0.523, 0.566)	0.021 (0.021, 0.021)	<.0001
127	0.45	0.54 (0.512, 0.567)	0.021 (0.021, 0.021)	<.0001
128	0.50	0.546 (0.52, 0.571)	0.021 (0.021, 0.021)	<.0001
129	0.55	0.531 (0.503, 0.559)	0.022 (0.021, 0.022)	<.0001
130	0.60	0.538 (0.513, 0.564)	0.022 (0.021, 0.022)	<.0001
131	0.65	0.533 (0.507, 0.559)	0.022 (0.022, 0.022)	<.0001
132	0.70	0.525 (0.496, 0.553)	0.022 (0.022, 0.022)	<.0001
133	0.75	0.525 (0.493, 0.557)	0.022 (0.022, 0.022)	<.0001
134	0.80	0.536 (0.501, 0.57)	0.022 (0.022, 0.023)	<.0001

135	0.85	0.538 (0.496, 0.581)	0.023 (0.022, 0.023)	<.0001
136	0.90	0.55 (0.503, 0.598)	0.023 (0.023, 0.023)	<.0001

Model group=4, Country x Sex: Fiji x Female

Scenario #	Quantile	Intercept	Height slope estimate	P-value
137	0.10	0.092 (-0.077, 0.262)	0.024 (0.023, 0.025)	<.0001
138	0.15	0.012 (-0.127, 0.151)	0.025 (0.024, 0.026)	<.0001
139	0.20	-0.044 (-0.157, 0.068)	0.025 (0.025, 0.026)	<.0001
140	0.25	-0.098 (-0.227, 0.03)	0.026 (0.025, 0.027)	<.0001
141	0.30	-0.109 (-0.221, 0.003)	0.026 (0.025, 0.027)	<.0001
142	0.35	-0.143 (-0.279, -0.007)	0.027 (0.026, 0.028)	<.0001
143	0.40	-0.155 (-0.276, -0.033)	0.027 (0.026, 0.028)	<.0001
144	0.45	-0.204 (-0.322, -0.086)	0.028 (0.027, 0.028)	<.0001
145	0.50	-0.253 (-0.335, -0.17)	0.028 (0.028, 0.029)	<.0001
146	0.55	-0.246 (-0.349, -0.143)	0.028 (0.028, 0.029)	<.0001
147	0.60	-0.242 (-0.336, -0.147)	0.028 (0.028, 0.029)	<.0001
148	0.65	-0.281 (-0.37, -0.192)	0.029 (0.028, 0.029)	<.0001
149	0.70	-0.311 (-0.423, -0.199)	0.029 (0.029, 0.03)	<.0001
150	0.75	-0.347 (-0.456, -0.238)	0.03 (0.029, 0.031)	<.0001
151	0.80	-0.382 (-0.509, -0.255)	0.03 (0.029, 0.031)	<.0001
152	0.85	-0.309 (-0.511, -0.107)	0.03 (0.029, 0.031)	<.0001
153	0.90	-0.157 (-0.429, 0.115)	0.03 (0.028, 0.031)	<.0001

Model group=4, Country x Sex: Fiji x Male

Scenario #	Quantile	Intercept	Height slope estimate	P-value
154	0.10	0.458 (0.4, 0.517)	0.021 (0.021, 0.021)	<.0001
155	0.15	0.487 (0.403, 0.57)	0.021 (0.021, 0.022)	<.0001
156	0.20	0.458 (0.378, 0.538)	0.021 (0.021, 0.022)	<.0001
157	0.25	0.403 (0.346, 0.46)	0.022 (0.022, 0.022)	<.0001
158	0.30	0.368 (0.292, 0.445)	0.022 (0.022, 0.023)	<.0001
159	0.35	0.352 (0.284, 0.42)	0.023 (0.022, 0.023)	<.0001
160	0.40	0.318 (0.236, 0.401)	0.023 (0.022, 0.023)	<.0001
161	0.45	0.301 (0.23, 0.372)	0.023 (0.023, 0.024)	<.0001
162	0.50	0.277 (0.2, 0.355)	0.024 (0.023, 0.024)	<.0001
163	0.55	0.254 (0.184, 0.323)	0.024 (0.023, 0.024)	<.0001
164	0.60	0.19 (0.099, 0.282)	0.024 (0.024, 0.025)	<.0001
165	0.65	0.178 (0.092, 0.263)	0.025 (0.024, 0.025)	<.0001
166	0.70	0.178 (0.069, 0.287)	0.025 (0.024, 0.026)	<.0001
167	0.75	0.173 (0.065, 0.28)	0.025 (0.024, 0.026)	<.0001
168	0.80	0.184 (0.089, 0.278)	0.025 (0.025, 0.026)	<.0001
169	0.85	0.21 (0.081, 0.34)	0.025 (0.024, 0.026)	<.0001
170	0.90	0.229 (0.084, 0.374)	0.026 (0.025, 0.026)	<.0001

Model group=4, Country x Sex: Haiti x Female

Scenario #	Quantile	Intercept	Height slope estimate	P-value
171	0.10	0.471 (0.415, 0.528)	0.021 (0.02, 0.021)	<.0001
172	0.15	0.441 (0.377, 0.505)	0.021 (0.021, 0.022)	<.0001
173	0.20	0.42 (0.367, 0.472)	0.022 (0.021, 0.022)	<.0001
174	0.25	0.409 (0.353, 0.464)	0.022 (0.021, 0.022)	<.0001
175	0.30	0.394 (0.333, 0.454)	0.022 (0.022, 0.023)	<.0001
176	0.35	0.368 (0.306, 0.431)	0.022 (0.022, 0.023)	<.0001
177	0.40	0.331 (0.275, 0.386)	0.023 (0.022, 0.023)	<.0001

178	0.45	0.284 (0.211, 0.357)	0.023 (0.023, 0.024)	<.0001
179	0.50	0.268 (0.207, 0.329)	0.024 (0.023, 0.024)	<.0001
180	0.55	0.241 (0.17, 0.311)	0.024 (0.023, 0.024)	<.0001
181	0.60	0.226 (0.155, 0.296)	0.024 (0.024, 0.025)	<.0001
182	0.65	0.182 (0.115, 0.248)	0.025 (0.024, 0.025)	<.0001
183	0.70	0.141 (0.058, 0.223)	0.025 (0.025, 0.026)	<.0001
184	0.75	0.122 (0.043, 0.201)	0.026 (0.025, 0.026)	<.0001
185	0.80	0.121 (0.046, 0.196)	0.026 (0.025, 0.026)	<.0001
186	0.85	0.118 (0.005, 0.232)	0.026 (0.025, 0.027)	<.0001
187	0.90	0.195 (0.077, 0.313)	0.026 (0.025, 0.027)	<.0001

Model group=4, Country x Sex: Haiti x Male

Scenario #	Quantile	Intercept	Height slope estimate	P-value
188	0.10	0.728 (0.679, 0.777)	0.019 (0.019, 0.019)	<.0001
189	0.15	0.732 (0.683, 0.781)	0.019 (0.019, 0.019)	<.0001
190	0.20	0.705 (0.666, 0.745)	0.019 (0.019, 0.02)	<.0001
191	0.25	0.708 (0.67, 0.747)	0.019 (0.019, 0.02)	<.0001
192	0.30	0.702 (0.664, 0.74)	0.02 (0.019, 0.02)	<.0001
193	0.35	0.702 (0.659, 0.745)	0.02 (0.019, 0.02)	<.0001
194	0.40	0.699 (0.66, 0.737)	0.02 (0.02, 0.02)	<.0001
195	0.45	0.707 (0.667, 0.747)	0.02 (0.02, 0.02)	<.0001
196	0.50	0.696 (0.652, 0.739)	0.02 (0.02, 0.02)	<.0001
197	0.55	0.705 (0.668, 0.742)	0.02 (0.02, 0.02)	<.0001
198	0.60	0.711 (0.673, 0.748)	0.02 (0.02, 0.02)	<.0001
199	0.65	0.701 (0.657, 0.746)	0.02 (0.02, 0.021)	<.0001
200	0.70	0.699 (0.653, 0.745)	0.02 (0.02, 0.021)	<.0001
201	0.75	0.695 (0.649, 0.74)	0.021 (0.02, 0.021)	<.0001
202	0.80	0.682 (0.629, 0.734)	0.021 (0.021, 0.021)	<.0001
203	0.85	0.677 (0.611, 0.744)	0.021 (0.021, 0.022)	<.0001
204	0.90	0.688 (0.595, 0.782)	0.021 (0.021, 0.022)	<.0001

Model group=4, Country x Sex: India x Female

Scenario #	Quantile	Intercept	Height slope estimate	P-value
205	0.10	0.545 (0.463, 0.628)	0.02 (0.019, 0.02)	<.0001
206	0.15	0.545 (0.493, 0.598)	0.02 (0.02, 0.021)	<.0001
207	0.20	0.522 (0.467, 0.577)	0.021 (0.02, 0.021)	<.0001
208	0.25	0.565 (0.482, 0.649)	0.021 (0.02, 0.021)	<.0001
209	0.30	0.535 (0.471, 0.599)	0.021 (0.021, 0.021)	<.0001
210	0.35	0.58 (0.505, 0.655)	0.021 (0.02, 0.021)	<.0001
211	0.40	0.598 (0.512, 0.684)	0.021 (0.02, 0.022)	<.0001
212	0.45	0.632 (0.573, 0.691)	0.021 (0.02, 0.021)	<.0001
213	0.50	0.634 (0.578, 0.69)	0.021 (0.021, 0.021)	<.0001
214	0.55	0.678 (0.603, 0.754)	0.021 (0.02, 0.021)	<.0001
215	0.60	0.664 (0.586, 0.742)	0.021 (0.021, 0.022)	<.0001
216	0.65	0.706 (0.622, 0.79)	0.021 (0.021, 0.022)	<.0001
217	0.70	0.747 (0.644, 0.85)	0.021 (0.02, 0.022)	<.0001
218	0.75	0.829 (0.691, 0.967)	0.021 (0.02, 0.022)	<.0001
219	0.80	0.894 (0.759, 1.029)	0.021 (0.02, 0.021)	<.0001
220	0.85	0.929 (0.765, 1.092)	0.021 (0.019, 0.022)	<.0001
221	0.90	1.159 (0.91, 1.409)	0.019 (0.018, 0.021)	<.0001

Model group=4, Country x Sex: India x Male

Scenario #	Quantile	Intercept	Height slope estimate	P-value
222	0.10	0.534 (0.472, 0.596)	0.02 (0.019, 0.02)	<.0001
223	0.15	0.564 (0.513, 0.614)	0.02 (0.019, 0.02)	<.0001
224	0.20	0.576 (0.53, 0.622)	0.02 (0.02, 0.02)	<.0001
225	0.25	0.567 (0.513, 0.62)	0.02 (0.02, 0.021)	<.0001
226	0.30	0.563 (0.509, 0.617)	0.02 (0.02, 0.021)	<.0001
227	0.35	0.568 (0.523, 0.612)	0.021 (0.02, 0.021)	<.0001
228	0.40	0.557 (0.513, 0.601)	0.021 (0.02, 0.021)	<.0001
229	0.45	0.554 (0.506, 0.601)	0.021 (0.021, 0.021)	<.0001
230	0.50	0.569 (0.516, 0.623)	0.021 (0.021, 0.021)	<.0001
231	0.55	0.549 (0.506, 0.593)	0.021 (0.021, 0.022)	<.0001
232	0.60	0.555 (0.488, 0.622)	0.021 (0.021, 0.022)	<.0001
233	0.65	0.576 (0.526, 0.626)	0.021 (0.021, 0.022)	<.0001
234	0.70	0.591 (0.535, 0.648)	0.022 (0.021, 0.022)	<.0001
235	0.75	0.6 (0.538, 0.661)	0.022 (0.021, 0.022)	<.0001
236	0.80	0.665 (0.577, 0.753)	0.021 (0.021, 0.022)	<.0001
237	0.85	0.714 (0.601, 0.828)	0.021 (0.021, 0.022)	<.0001
238	0.90	0.9 (0.718, 1.082)	0.021 (0.019, 0.022)	<.0001

Model group=4, Country x Sex: Indonesia x Female

Scenario #	Quantile	Intercept	Height slope estimate	P-value
239	0.10	0.554 (0.47, 0.639)	0.02 (0.019, 0.02)	<.0001
240	0.15	0.542 (0.475, 0.609)	0.02 (0.02, 0.021)	<.0001
241	0.20	0.529 (0.448, 0.609)	0.02 (0.02, 0.021)	<.0001
242	0.25	0.524 (0.476, 0.573)	0.021 (0.02, 0.021)	<.0001
243	0.30	0.495 (0.444, 0.545)	0.021 (0.021, 0.021)	<.0001
244	0.35	0.481 (0.438, 0.524)	0.021 (0.021, 0.022)	<.0001
245	0.40	0.462 (0.408, 0.515)	0.021 (0.021, 0.022)	<.0001
246	0.45	0.475 (0.404, 0.545)	0.021 (0.021, 0.022)	<.0001
247	0.50	0.445 (0.375, 0.514)	0.022 (0.021, 0.022)	<.0001
248	0.55	0.435 (0.38, 0.491)	0.022 (0.022, 0.022)	<.0001
249	0.60	0.412 (0.35, 0.473)	0.022 (0.022, 0.023)	<.0001
250	0.65	0.415 (0.349, 0.481)	0.022 (0.022, 0.023)	<.0001
251	0.70	0.399 (0.312, 0.485)	0.023 (0.022, 0.023)	<.0001
252	0.75	0.423 (0.318, 0.529)	0.023 (0.022, 0.023)	<.0001
253	0.80	0.459 (0.354, 0.564)	0.023 (0.022, 0.023)	<.0001
254	0.85	0.472 (0.342, 0.603)	0.023 (0.022, 0.024)	<.0001
255	0.90	0.492 (0.332, 0.652)	0.023 (0.022, 0.024)	<.0001

Model group=4, Country x Sex: Indonesia x Male

Scenario #	Quantile	Intercept	Height slope estimate	P-value
256	0.10	0.536 (0.478, 0.594)	0.02 (0.02, 0.02)	<.0001
257	0.15	0.558 (0.503, 0.614)	0.02 (0.02, 0.02)	<.0001
258	0.20	0.569 (0.524, 0.613)	0.02 (0.02, 0.02)	<.0001
259	0.25	0.582 (0.536, 0.627)	0.02 (0.02, 0.02)	<.0001
260	0.30	0.589 (0.547, 0.631)	0.02 (0.02, 0.02)	<.0001
261	0.35	0.592 (0.554, 0.629)	0.02 (0.02, 0.02)	<.0001
262	0.40	0.597 (0.557, 0.636)	0.02 (0.02, 0.021)	<.0001
263	0.45	0.599 (0.564, 0.634)	0.02 (0.02, 0.021)	<.0001
264	0.50	0.61 (0.566, 0.655)	0.02 (0.02, 0.021)	<.0001
265	0.55	0.603 (0.569, 0.637)	0.02 (0.02, 0.021)	<.0001
266	0.60	0.613 (0.572, 0.654)	0.02 (0.02, 0.021)	<.0001
267	0.65	0.612 (0.561, 0.664)	0.021 (0.02, 0.021)	<.0001

268	0.70	0.627 (0.578, 0.676)	0.021 (0.02, 0.021)	<.0001
269	0.75	0.634 (0.587, 0.681)	0.021 (0.02, 0.021)	<.0001
270	0.80	0.634 (0.589, 0.679)	0.021 (0.021, 0.021)	<.0001
271	0.85	0.633 (0.585, 0.681)	0.021 (0.021, 0.021)	<.0001
272	0.90	0.661 (0.55, 0.773)	0.021 (0.02, 0.022)	<.0001

Model group=4, Country x Sex: PNG x Female

Scenario #	Quantile	Intercept	Height slope estimate	P-value
273	0.10	0.393 (0.322, 0.464)	0.022 (0.021, 0.022)	<.0001
274	0.15	0.386 (0.289, 0.482)	0.022 (0.021, 0.023)	<.0001
275	0.20	0.371 (0.299, 0.443)	0.022 (0.022, 0.023)	<.0001
276	0.25	0.373 (0.297, 0.449)	0.023 (0.022, 0.023)	<.0001
277	0.30	0.407 (0.336, 0.479)	0.022 (0.022, 0.023)	<.0001
278	0.35	0.397 (0.305, 0.49)	0.023 (0.022, 0.023)	<.0001
279	0.40	0.406 (0.309, 0.503)	0.023 (0.022, 0.023)	<.0001
280	0.45	0.42 (0.347, 0.494)	0.023 (0.022, 0.023)	<.0001
281	0.50	0.413 (0.314, 0.512)	0.023 (0.022, 0.024)	<.0001
282	0.55	0.457 (0.351, 0.563)	0.023 (0.022, 0.024)	<.0001
283	0.60	0.475 (0.377, 0.574)	0.023 (0.022, 0.023)	<.0001
284	0.65	0.482 (0.396, 0.568)	0.023 (0.022, 0.023)	<.0001
285	0.70	0.513 (0.398, 0.628)	0.023 (0.022, 0.024)	<.0001
286	0.75	0.533 (0.429, 0.638)	0.023 (0.022, 0.024)	<.0001
287	0.80	0.503 (0.371, 0.635)	0.023 (0.022, 0.024)	<.0001
288	0.85	0.525 (0.353, 0.698)	0.023 (0.022, 0.024)	<.0001
289	0.90	0.662 (0.351, 0.972)	0.023 (0.021, 0.025)	<.0001

Model group=4, Country x Sex: PNG x Male

Scenario #	Quantile	Intercept	Height slope estimate	P-value
290	0.10	0.585 (0.528, 0.641)	0.021 (0.02, 0.021)	<.0001
291	0.15	0.564 (0.496, 0.632)	0.021 (0.02, 0.021)	<.0001
292	0.20	0.577 (0.523, 0.631)	0.021 (0.021, 0.021)	<.0001
293	0.25	0.601 (0.56, 0.642)	0.021 (0.021, 0.021)	<.0001
294	0.30	0.598 (0.536, 0.661)	0.021 (0.021, 0.022)	<.0001
295	0.35	0.629 (0.578, 0.679)	0.021 (0.021, 0.021)	<.0001
296	0.40	0.658 (0.598, 0.719)	0.021 (0.021, 0.021)	<.0001
297	0.45	0.644 (0.591, 0.697)	0.021 (0.021, 0.021)	<.0001
298	0.50	0.653 (0.598, 0.707)	0.021 (0.021, 0.022)	<.0001
299	0.55	0.665 (0.611, 0.719)	0.021 (0.021, 0.022)	<.0001
300	0.60	0.699 (0.632, 0.765)	0.021 (0.021, 0.022)	<.0001
301	0.65	0.715 (0.647, 0.783)	0.021 (0.021, 0.022)	<.0001
302	0.70	0.743 (0.683, 0.803)	0.021 (0.021, 0.021)	<.0001
303	0.75	0.785 (0.716, 0.854)	0.021 (0.02, 0.021)	<.0001
304	0.80	0.819 (0.717, 0.921)	0.021 (0.02, 0.021)	<.0001
305	0.85	0.925 (0.76, 1.089)	0.02 (0.019, 0.021)	<.0001
306	0.90	1.075 (0.875, 1.274)	0.019 (0.018, 0.021)	<.0001
